# Differential metal-binding properties of dynamic acylhydrazone polymers and their sensing applications

**DOI:** 10.1098/rsos.170466

**Published:** 2017-08-30

**Authors:** Siheng Gao, Lijie Li, Ismail Vohra, Daijun Zha, Lei You

**Affiliations:** 1 State Key Laboratory of Structural Chemistry, Fujian Institute of Research on the Structure of Matter, Chinese Academy of Sciences, Fuzhou 350002, People's Republic of China; 2 University of Chinese Academy of Sciences, Beijing 100049, People's Republic of China

**Keywords:** polyacylhydrazone, metal-binding properties, responsive polymers, sensing

## Abstract

As one of common dynamic covalent bonds, acylhydrazone bond plays an important role in developing intelligent responsive materials. In this report, we present acylhydrazone-based dynamic polymers with multi-stimuli responsiveness, particularly metal recognition behaviours and their modulation. A series of polyacylhydrazones with different metal-binding sites were designed and prepared in a modular fashion. Titration of these receptors with a diverse set of metal ions, including Cu^2+^, Zn^2+^ and La^3+^, resulted in unique optical changes, and both the sensitivity and selectivity profiles can be regulated. Moreover, the metal-binding feature was facilely modulated by changing the solvent. The addition of weakly basic anions was employed to further fine-tune the responsiveness of the polymers by taking advantage of the cooperative effect with metal coordination. Finally, the sensitive detection of 6-mercaptopurine and pyrophosphate was achieved to demonstrate the application potential of these systems.

## 1. Introduction

Stimuli-responsive materials have potential applications in the research of biointerfaces, [1] optical sensing and imaging, [2,3] gas storage and transformation, [4] tissue engineering, [5] as well as drug and gene delivery [6,7]. Thus, the investigation of these intelligent materials has garnered significant attention over the past decades [8–11]. One notable strategy for the construction of responsive materials is the use of dynamic covalent bonds, such as imine [12,13] and disulfide [14,15]. The field of dynamic covalent chemistry has been growing rapidly, and the reversible formation and exchange of dynamic covalent bonds can provide excellent opportunities for the manipulation of properties, such as responsiveness, adaptability and self-healing [16–19].

Acylhydrazone, created through the condensation of carbonyl and acylhydrazide, is one of the most employed reversible covalent bonds [20–22]. Its high stability in water in conjunction with the presence of molecular recognition sites, such as hydrogen bonding and metal coordination sites, makes the acylhydrazone an ideal platform for the development of intelligent materials [23–28]. In particular, the strategy of subcomponent self-assembly was used for the creation and regulation of polyacylhydrazone structure. For instance, Lehn's group showed that polyacylhydrazones are able to undergo the recombination of their components at ordinary temperature with acid catalyst [29]. Deng's group prepared acylhydrazone-based novel dynamic covalent gels exhibiting self-healing feature [30]. Moreover, Sanders [31] reported a linear acylhydrazone oligomer for the recognition of dihydrogen phosphate. In addition, a variety of hydrazone-derived chemosensors were designed to detect specific analyte bearing great selectivity and sensitivity [32–36].

Pyridine-2-carboxyaldehyde, [37] salicylaldehyde [38,39] and their corresponding derivatives are widespread building blocks in imine-based assemblies, including metal-organic cages [40–42] and covalent-organic frameworks [43–45]. However, their dynamic hydrazone assemblies have received significantly less attention. We conceived that the combination of the intrinsic recognition motifs of acylhydrazone unit and additional metal coordination sites from the aldehyde would afford us a facile and versatile platform for modulating the differential metal-binding properties. Although metal complexes of salicylaldehyde hydrazones have been previously studied, [46,47] they have been rarely explored within the context of dynamic polymers. Herein, we present a systematic study of acylhydrazone polymers with distinct metal-binding profiles, which were further modulated by external stimuli, including solvents and anions. Furthermore, two model analytes were detected by using these polymers.

## 2. Results and discussion

### 2.1. Design and synthesis

**Scheme 1.**
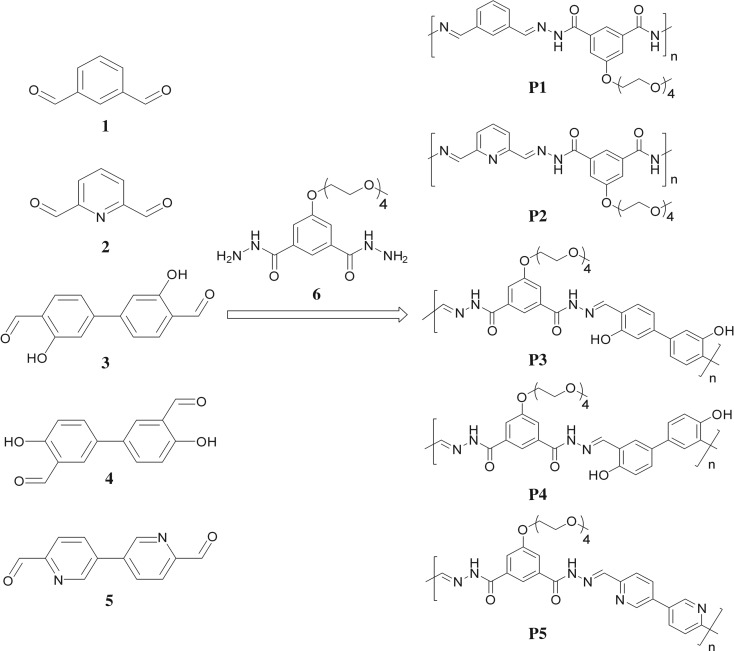
Chemical structure of the monomers and the associated polymers.

In order to fine-tune the metal-binding properties of acylhydrazone polymers, monomers with different binding sites (**1**–**5**), such as basic pyridine and acidic phenol, were chosen (scheme 1). As pyridine-2-carboxyaldehyde derivatives, pyridine-2,6-dicarbaldehyde (**2**) and [3,3′]bipyridinyl-6,6′-dicarbaldehyde (**5**) were used. Two salicylaldehyde derivatives with opposite substitution pattern were employed: 3,3′-dihydroxy-biphenyl-4,4′-dicarbaldehyde (**3**) and 4,4′-dihydroxy-biphenyl-3,3′-dicarbaldehyde (**4**). One control aldehyde (benzene-1,3-dicarbaldehyde, **1**) was also studied. A seven-step synthetic sequence for **3** was designed as outlined in the electronic supplementary material, scheme S1. As shown in scheme 1, the assembly of the acylhydrazone polymers (**P1** to **P5**) involved a 1 : 1 mixture of dihydrazides and dialdehydes building blocks. One common dihydrazide containing an oligo (ethylene glycol) chain, which aims to improve the solubility of the polymers, was chosen.

### 2.2. Polymers characterization

**Figure 1.**
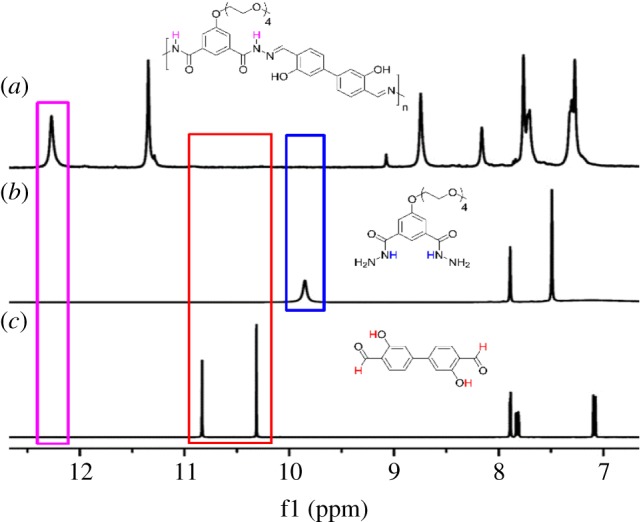
^1^H-NMR of (*a*) P3, as well as the corresponding (*b*) dihydrazide 6 and (*c*) dialdehyde 3 in DMSO-*d*_6._

To our delight, these polymers have good solubility in solvents, such as DMSO and DMF, and they were all characterized by ^1^H-NMR and GPC spectroscopy, respectively. Taking **P3** as an example, ^1^H-NMR signal around 10.2 ppm (figure 1*c*) which was assigned to dialdehyde, and the resonance around 9.8 ppm (figure 1*b*), which was assigned to dihydrazide, disappeared, while a new signal at 12.2 ppm (figure 1*a*) emerged, indicating the formation of acylhydrazone linkages. GPC results also show the successful formation of the desired polymer (Mw = 22407, Mn = 13294, PDI = 1.69; electronic supplementary material, figure S23). Other polymers were analysed in a similar fashion, and the corresponding data of **P1**, **P2** and **P4** is listed in the electronic supplementary material, figures S21, S22 and S24.

**Figure 2.**
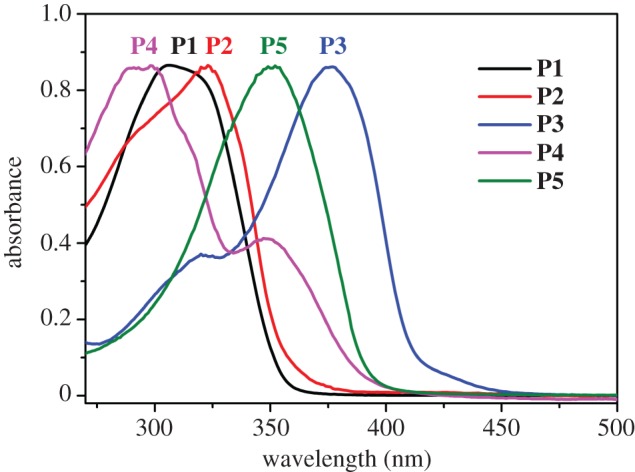
UV-vis spectra of polymers **P1**-**P5**.

In addition, UV-vis spectroscopy was measured to further understand the structure and optical properties of the polymers. As shown in figure 2, the absorbance spectra of **P1** and **P2**, for which the aldehyde moiety varied from benzene to pyridine, were slightly different. By contrast to **P2**, **P5** had a larger pi-system with bipyridine embedding in the polymer backbone, leading to a red shift of 35 nm. For **P3**, a further bathochromic shift around 30 nm was observed. Such an effect is probably due to the extension of conjugation as a result of the electron-donating ability of the hydroxyl group through resonance. It was interesting to note that the absorption spectra of **P3** and **P4**, for which the only difference is the placement of C=N and OH, were quite different, with **P4** affording the smallest maximum absorption wavelength while **P3** gave the largest (the gap was around 80 nm). These results appeared to reveal that the structure of **P4** was twisted while **P3** had a more coplanar arrangement. The varied structures and optical signals of these polyacylhydrazones paved the way for the control of metal binding.

### 2.3. Metal ions binding in DMSO

**Figure 3.**
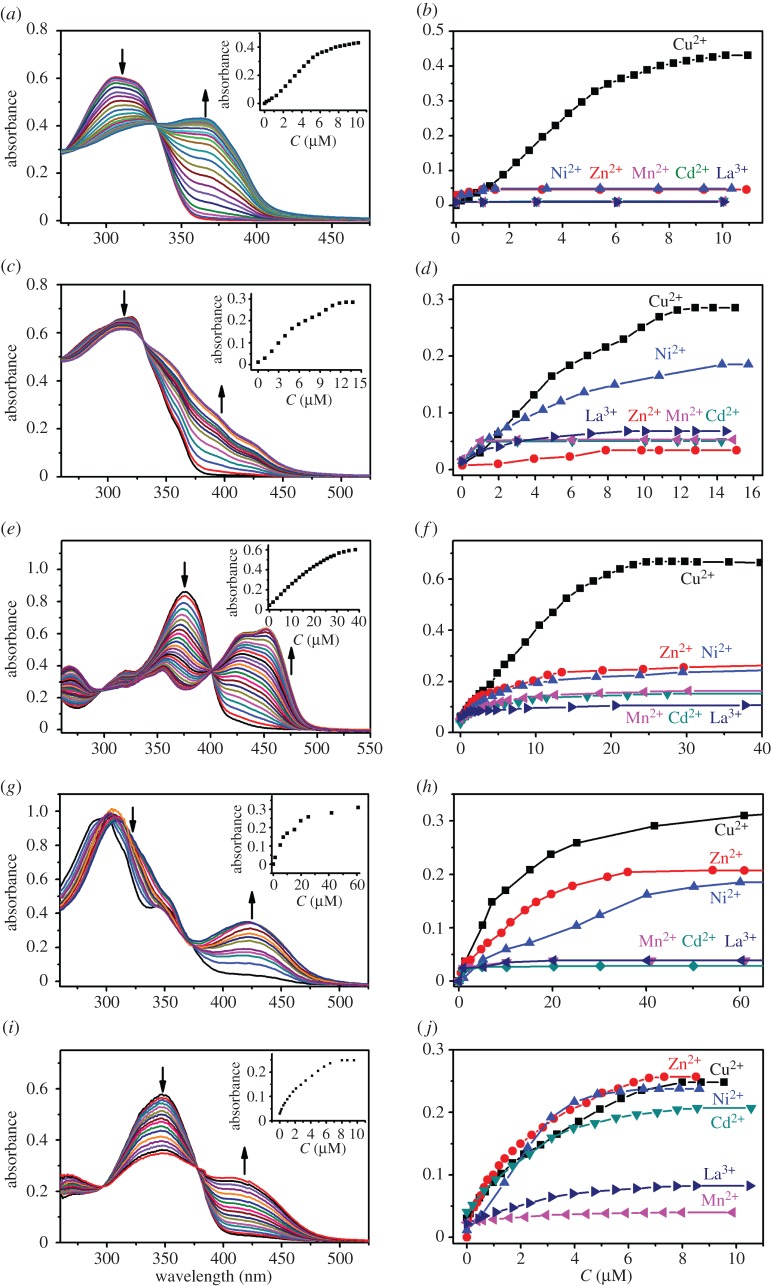
UV-vis spectra of **P1**-**P5** upon titration of Cu(OTf)_2_ in DMSO. (*a*) **P1** (15.2 µg ml^−1^), Cu(OTf)_2_ (0–10 µM); (*c*) **P2** (17.8 µg ml^−1^), Cu(OTf)_2_ (0–14 µM); (*e*) **P3** (7.97 µg ml^−1^), Cu(OTf)_2_ (0–40 µM); (*g*) **P4** (9.37 µg ml^−1^), Cu(OTf)_2_ (0–60 µM); (*i*) **P5** (10.6 µg ml^−1^), Cu(OTf)_2_ (0–10 µM). UV-vis titration isotherm of (*b*) **P1**; (d) **P2**; (f) **P3**; (*h*) **P4** and (*j*) **P5** upon addition of various of metal cations. Inset: absorbance changes with the addition of corresponding cations.

With acylhydrazone-based polymers in hand, their responses towards metal ions were examined in detail. The UV-vis spectral changes of the polymers upon the titration with Cu(OTf)_2_ are shown in figure 3. With the stepwise addition of Cu^2+^ (0–10 µM) to the DMSO solution of **P1** (15.2 µg ml^−1^) which lacks coordination sites except acylhydrazone groups (i.e. a bidentate ligand) in the main chain, a new absorption band centred at 375 nm was found, while the peak at 320 nm decreased (figure 3*a*). The results suggest that copper coordination occurred, and a stable complex formed, which led to enhanced coplanarity of the polymer and hence a bathochromic shift. Moreover, one clear isosbestic point at 327 nm appeared, which indicates that all the binding sites along the polymer chains coordinate Cu^2+^ in a similar way. Absorption spectra were next recorded with other metal ions, such as Zn^2+^, Ni^2+^, Mn^2+^, Cd^2+^ and La^3+^ (see the electronic supplementary material), and they exhibited very slightly or almost no absorbance change at 375 nm. The comparison of titration isotherms (figure 3*b*) further confirms that **P1** afforded a significant Cu^2+^-triggered spectral response with great selectivity.

To facilitate the metal chelation, polymers bearing tridentate acylhydrazone units (**P2**--**P5**) were studied. As would be expected, **P2** gave the responses to Ni^2+^ and Cu^2+^ among the aforementioned metal ions (figure 3*c*,*d*). A bathochromic shift was also detected, analogous to **P1**. However, there was no new absorbance maximum occurring apparently even though an increase around 400 nm indeed occurred, and the decrease of the original peak at 325 nm was only modest. We rationalize these observations with the distortion of the polymer backbone. To alleviate this concern, **P5** with bipyridine units was studied further (figure 3*i*,*j*). Gratifyingly, both the decrease at 350 nm and the increase at 425 nm were pronounced, despite that the selectivity was compromised again. This is reasonable because the rotation about C–C bond between two pyridines increases the capability of metal binding with minimal distortion of the polymer chain. Two phenol-containing polymers (**P3** and **P4**) were finally probed. Although both of them showed obvious responses to Cu^2+^, the optical changes of **P3** were much more significant than **P4** (figure 3*e*,*g*), and **P3** also exhibited better selectivity for Cu^2+^ than **P4** (figure 3*f*,*h*). In all, through the introduction of aldehyde motif with varied structural and molecular recognition feature, both the magnitude of metal-induced optical responses and selectivity profiles can be readily regulated.

### 2.4. Solvent effect

**Figure 4.**
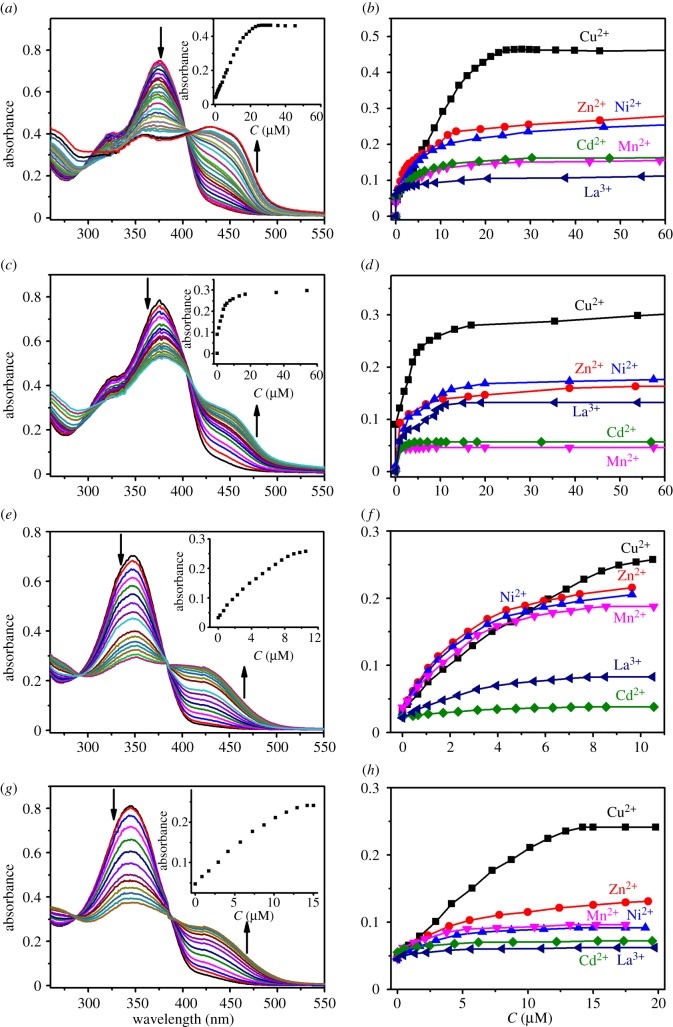
(*a*) UV-vis absorption spectra of **P3** (7.97 µg ml^−1^) upon addition of Cu(OTf)_2_ (0–60 µM) and (*b*) the corresponding titration isotherm of **P3** upon addition of various metal cations in 4 : 1 DMSO/H_2_O solution; (*c,d*) UV-vis absorption spectra and corresponding titration isotherm of **P3** (7.97 µg ml^−1^) in 1 : 1 DMSO/H_2_O solution; (*e*,*g*,*f,h*) UV-vis absorption spectra and corresponding titration isotherm of **P5** (10.6 µg ml^−1^) in the similar fashion as **P3**. Inset: absorbance changes with the addition of corresponding cations.

The coordination sphere of metal centres can be altered significantly through the use of different solvent (i.e. solvation), and hence, solvent effect was explored as a means of modulating metal binding. Towards this end, **P3** and **P5** were chosen depending on their basic (pyridine) and acidic (phenol) residues, respectively, and their solvent effects were contrasted. Furthermore, considering the solubility of the polymers 4 : 1 and 1 : 1 mixtures of DMSO and H_2_O were employed. With the increase of the percentage of water in DMSO, the absorbance of **P3** at 450 nm in the presence of Cu^2+^ became weaker (figure 4*a*,*c*). The solution of **P3** with other metal cations (Mn^2+^, Ni^2+^, Zn^2+^, Cd^2+^ and La^3+^) still exhibited smaller absorbance changes at 450 nm compared with Cu^2+^ (figure 4*b*,*d*). However, the discrimination between Cu^2+^ and other metal ions became worse relative to data in DMSO (figure 3*b*). The hydration of metal ions leads to poorer response as a result of the competition between water and polymers for metal ions. Concomitantly, water facilitates the deprotonation of phenol OH, thus giving inconspicuous discrimination between metal ions. Similarly, there was a decrease in absorbance of **P5** at 425 nm upon the addition of Cu^2+^ in aqueous solution (figure 4*e*,*g*). However, better selectivity for Cu^2+^ was observed, especially in 1 : 1 DMSO/H_2_O (figure 4*f*,*h*), which was consistent with the previous work [48]. As a result, both the sensitivity and selectivity of these polymers towards metal ions can be modulated by changing the solvent.

### 2.5. Cooperative effect

**Figure 5.**
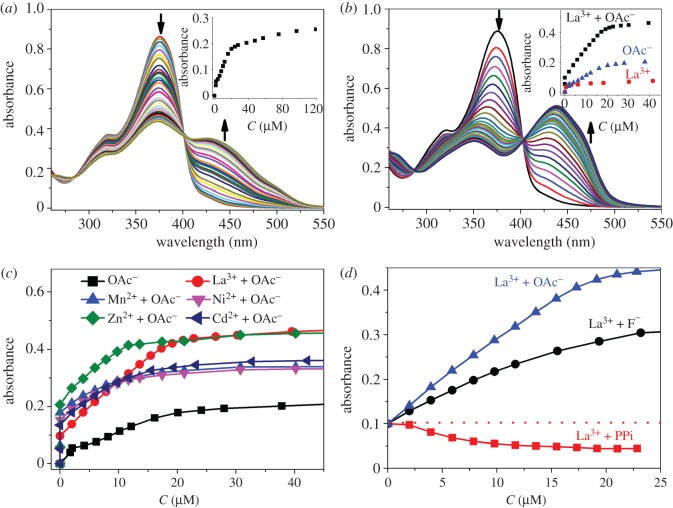
(*a*) UV-vis absorption spectra of **P3** (7.97 µg ml^−1^) upon addition of Bu_4_NOAc (0–120 µM) in DMSO; (*b*) UV-vis absorption spectra of **P3** (7.97 µg ml^−1^) upon addition of Bu_4_NOAc (0–45 µM) in the presence of La(OTf)_3_ (15.4 µM) in DMSO; (*c*) UV-vis titration isotherm of **P3** (7.97 µg ml^−1^) upon addition of Bu_4_NOAc in the presence of various metal cations and (*d*) UV-vis titration isotherm of **P3** (7.97 µgml^−1^) upon addition of various anions in the presence of La(OTf)_3_ (15.4 µM) in DMSO. Inset: absorbance changes with the addition of corresponding cations or anions.

We next set out to diversify the responsiveness of our systems. The NH within acylhydrazone group and phenol OH are weakly acidic. Therefore, the acid/base equilibrium between **P3** and external anions exhibiting varied basicity can offer abundant opportunities for dictating stimuli-responsive properties. Taking into account these considerations, UV-vis spectra of **P3** were firstly measured in the presence of tetrabutylammonium salts of different anions (see the electronic supplementary material), including F^−^, Cl^−^, Br^−^, OAc^−^, trifluomethanesulfonate (OTf^−^) and hydrogen pyrophosphate (PPi). The absorption band at 375 nm decreased with an increase in the peak around 450 nm when OAc^−^ was added (figure 5*a*). Similar behaviour was also observed for basic F^−^ (electronic supplementary material, figure S57) and PPi (electronic supplementary material, figure S58), though the extent of signal changes was anion-dependent. The optical changes upon addition of Cl^−^, Br^−^ and OTf^−^ had not been observed (electronic supplementary material, figures S59–S61). We interpret the optical changes of **P3** towards weakly basic anions with the deprotonation of acylhydrazone NH and/or phenol OH, resulting in enhanced conjugation and thereby a red shift. These results are also consistent with the pH effect studied in our previous work [48].

Since both metal ions and anions are able to induce differential optical responsiveness of polymeric receptors, attention was then turned to their cooperative effects in order to further fine-tune the system. Towards this end, a pair of OAc^−^ and La^3+^ was selected as a proof-of-concept study. Slight changes were observed during the titration of La(OTf)_3_ into a solution of **P3** in DMSO (electronic supplementary material, figure S56). By contrast, in the presence of La^3+^, there was a significant increase in the absorbance at 450 nm upon the addition of OAc^−^ (figure 5*b*), indicative of a positive cooperative effect. Similar signal enhancement was also observed for other metal ions, such as Mn^2+^, Ni^2+^, Zn^2+^ and Cd^2+^ (electronic supplementary material, figures S95–S98), as evidenced by the comparison of titration isotherms of OAc^−^ with or without metal ions (figure 5*c*). Such a positive cooperative effect is rationalized by the facilitation of metal binding through anion-induced deprotonation. This mechanism is also verified through the pairing of F^−^ and La^3+^ (figure 5*d*). Interestingly, the absorption at 450 nm abated when PPi was added into a solution of **P3** in the presence of La(OTf)_3_ (figure 5*d*). One explanation comes from the displacement of **P3** by PPi as PPi has a better affinity for La^3+^. In other words, a competition between **P3** and PPi for the coordination of La^3+^ dominated.

### 2.6. Sensing applications

**Figure 6.**
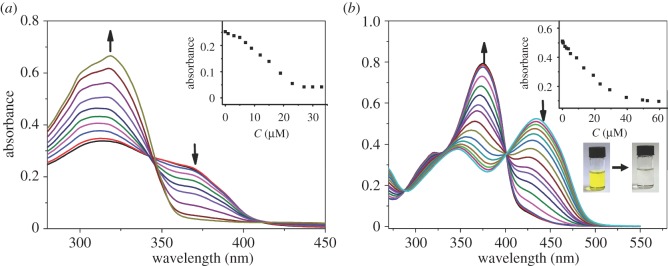
(*a*) UV-vis of **P1** (15.2 µg ml^−1^) solution upon titration with 6-MP (0–32 µM) in the presence of Cu^2+^ (9.8 µM) in DMSO; (*b*) UV-vis of **P3** (7.97 µg ml^−1^) solution with addition of PPi (0–60 µM) in the presence of La(OAc)_3_ (4.9 µM) in DMSO. Inset: absorbance changes with the addition of corresponding analytes.

After fine-tuning metal-binding properties of polyacylhydrazones, the sensing of 6-MP and PPi was explored as a proof of concept in order to demonstrate the application potential of these polymers. 6-mercaptopurine (6-MP, as shown in electronic supplementary material, figure S99) is a chemotherapy anti-cancer drug with immunosuppressant properties. Owing to its serious side effect and variable activity around the plasma concentration, it is of significance to develop a simple assay for 6-MP [49]. Inspired by its ability to coordinate with Cu^2+^, a sensing system for 6-MP was created based on the strategy of indicator displacement assay. For instance, with the stepwise addition of 6-MP to a solution of **P1** and Cu(OTf)_2_ in DMSO (figure 6*a*), the absorption band around 375 nm gradually disappeared with the concomitant recovery of the original peak of **P1** at 320 nm. These results are consistent with the replacement of **P1** by 6-MP in copper complexes. A limit of detection of 6-MP was revealed around 0.47 µM. Similar experiments were performed on **P2** (electronic supplementary material, figure S99) and **P3** (electronic supplementary material, figure S100). However, the displacement of Cu^2+^ by 6-MP did not occur. These results echo the stronger metal-binding abilities of **P2** or **P3** than **P1**. Furthermore, when a strong metal chelating agent (EDTA) was added to the above solution, Cu^2+^ was indeed replaced completely (electronic supplementary material, figures S101 and S102), thereby validating the sensing mechanism.

PPi is the product of ATP hydrolysis under cellular conditions, and it plays a significant role in energy storage and signal transduction [50]. On the basis of results described in figure 5, PPi was added to a solution of **P3** (7.97 µM) in the presence of La(OAc)_3_ (4.9 µM). As shown in figure 6*b*, **P3** was completely replaced by PPi. This method was used to detect PPi in the low micro molar range.

## 3. Conclusion

In summary, a series of acylhydrazone-based polymers with distinctive metal-binding sites were designed and synthesized. Differential metal ions binding properties were investigated in detail, and both the sensitivity and selectivity profiles can be regulated. The optical response of these polymers was further modulated by simply varying solvent and adding anions. Particularly, different mechanism (cooperative effect or competition) was involved in the latter case. By taking advantage of responsiveness feature, the sensitive detection of 6-MP and PPi was finally achieved to showcase the application potential of these systems.

## 4. Experimental section

### 4.1. Materials

Anhydrous methanol, anhydrous ethanol, dichloromethane, hydrazine hydrate, *N*,*N*-dimethylformamide and dimethyl sulfoxide were purchased from Sinopharm Chemical Reagent Co., Ltd. CDCl_3_ and DMSO-d_6_ were purchased from Aldrich. All the other reagents were obtained from commercial sources and were used without further purification, unless indicated otherwise.

### 4.2. Preparation of polymers

The general procedure for the preparation of polymers was described as follows: the dialdehyde and dihydrazide (0.2 mmol, the feed ratio is 1 : 1) were dissolved in methanol (15 ml). Under inert atmosphere, the mixture solution was stirred and refluxed for 1 day in the presence of catalytic amount of TFA. After cooling down to room temperature, the precipitate was filtered and washed thoroughly with hot MeOH. After drying under vacuum, the polymers were obtained as powder solids.

## Supplementary Material

Supplementary Information

## Data accessibility

All relevant data are within the paper and its electronic supplementary material files.
