# Supplementary material for: Differential metal-binding properties of dynamic acylhydrazone polymers and their sensing applications
Source: R Soc Open Sci. 2017 Aug 30;4(8):170466. doi: 10.1098/rsos.170466 (PMC5579109; doi:10.1098/rsos.170466)
Supplement: Supplementary Information [file rsos170466supp1.docx]

**Supplementary Information**

**Differential Metal Binding Properties of Dynamic Acylhydrazone Polymers and Their Sensing Applications**

Siheng Gao^1,2^, Lijie Li^1^, Ismail Vohra^1^, and Daijun Zha^1^ and Lei You^1^

1. State Key Laboratory of Structural Chemistry, Fujian Institute of Research on the Structure of Matter, Chinese Academy of Sciences, Fuzhou 350002, China.

2. University of Chinese of Academy of Sciences, Beijing 100049, China.

E-mail: [zhad@fjirsm.ac.cn](mailto:zhad@fjirsm.ac.cn)

[lyou@fjirsm.ac.cn](mailto:lyou@fjirsm.ac.cn)

Table of Content

[1. Experimental Section S3](#_Toc480807103)

[**1.1** **General Methods** S3](#_Toc480807104)

[**1.2** **Materials** S3](#_Toc480807105)

[**1.3** **Synthesis and Characterization** S3](#_Toc480807106)

[2. ^1^H-NMR and ^13^C-NMR S8](#_Toc480807108)

[3. GPC Data S18](#_Toc480807109)

[4. Spectroscopic Data S20](#_Toc480807110)

[**4.1.** **Polymer 1 in DMSO Solution** S20](#_Toc480807111)

[**4.2.** **Polymer 1 in DMSO : H_2_O (1 : 1 v/v) Solution** S23](#_Toc480807112)

[**4.3.** **Polymer 2 in DMSO Solution** S24](#_Toc480807113)

[**4.4.** **Polymer 2 in DMSO : H_2_O (1 : 1 v/v) Solution** S27](#_Toc480807114)

[**4.5.** **Polymer 3 in DMSO Solution** S29](#_Toc480807115)

[**4.6.** **Polymer 3 in DMSO : H_2_O (4 : 1 v/v) Solution** S32](#_Toc480807116)

[**4.7.** **Polymer 3 in DMSO : H_2_O (1 : 1 v/v) Solution** S35](#_Toc480807117)

[**4.8.** **Polymer 4 in DMSO Solution** S37](#_Toc480807118)

[**4.9.** **Polymer 5 in DMSO Solution** S39](#_Toc480807119)

[**4.10.** **Polymer 5 in DMSO : H_2_O (4 : 1 v/v) Solution** S40](#_Toc480807120)

[**4.11.** **Polymer 5 in DMSO : H_2_O (1 : 1 v/v) Solution** S42](#_Toc480807121)

[5. The Cooperative Effect S43](#_Toc480807122)

[6. Sensing Application S45](#_Toc480807123)

[7. Reference S48](#_Toc480807124)

1. **Experimental Section**
   1. **General Methods**

^1^H-NMR (400 MHz) and ^13^C-NMR (100 MHz) spectra were recorded with a Bruker Biospin avance III spectrometer at room temperature. The chemical shifts (δ) for ^1^H-NMR and ^13^C-NMR spectra, given in ppm, are referenced to the residual proton signal of the deuterated solvent (DMSO-*d_6_* or CDCl_3_).

Gel permeation chromatography was run on a Waters 1515 high performance liquid chromatography instrument equipped with a 2414 differential refractometer and a set of Styragel mixed-C columns (Styragel Waters HR4E and HR5E) to separate molecular weights ranging from 10^2^ to 10^6^. The oven temperature was set at 40 °C. THF was used as the eluent, and the flow rate was 1 mL/min. Monodispersed polystyrene standards (Aldrich Chemical Co.) were used to generate the calibration curve.

UV/Vis spectra were recorded on a Lambda 900 spectrophotometer. Deionized water and DMSO (HPLC of purity) were used for the titration. Each sample was allowed to equilibrate before the spectrum was recorded during the course of titration. The corresponding binding isotherm was generated.

- 1. **Materials**

Anhydrous methanol, anhydrous ethanol, dichloromethane, hydrazine hydrate, N,N-dimethylformamide, and dimethyl sulfoxide were purchased from Sinopharm Chemical Reagent Co., Ltd. CDCl_3_ and DMSO-*d_6_* were purchased from Aldrich. All the other reagents were obtained from commercial sources and were used without further purification, unless indicated otherwise.

- 1. **Synthesis and Characterization**

**Scheme S1**. The synthetic route of monomers

******: 4-Amino-2-hydroxybenzoic acid (3.1 g, 20 mmol) was suspended in H_2_SO_4_ (20 mL, 2.7 M) at -5 ^o^C. NaNO_2_ (1.4 g, 21 mmol) in H_2_O (5 mL) was cooled to ice bath temperature and was added dropwise to the above suspension over several minutes. The resulting mixture was stirred at -5 °C for 30 minutes. A solution of KI (5.0 g, 30 mmol) in H_2_SO_4_ (10 mL, 1 M) was added dropwise to the diazonium salt, with considerable evolution of N_2_. The mixture was heated at 70 ^o^C for 1 h. After cooling down, the mixture was partitioned between H_2_O and EtOAc. The organic layers were dried over Na_2_SO_4_. The residue was purified by column chromatography (MeOH/CH_2_Cl_2_ = 1:20) to afford a deep yellow solid (1.32 g, 25%). ^1^H-NMR (DMSO-*d*_6_) *δ* = 7.71 (d, *J* = 8.5 Hz, 1H), 7.22 (s, 1H), 7.13 (d, *J* = 8.5 Hz, 1H).

******: 2-hydroxy-4-iodobenzoic acid (1.0 g, 3.8 mmol) was dissolved in acetone (30 mL). Potassium carbonate (2.6 g, 19 mmol) and CH_3_I (2.5 g, 17.6 mmol) was added in sequence, and then the mixture was refluxed with stirring until the TLC indicated the starting material was consumed. After cooling down, the excess K_2_CO_3_ was filtered off and wash thoroughly with acetone. The solvent was concentrated, and water (10 mL) was added, and the aqueous layer was extracted with EtOAc, dried over Na_2_SO_4_, and evaporated. The residue was purified by column chromatography (petroleum ether/ethyl acetate = 10:1) to give a yellow oil (1.0 g, 90%). ^1^H-NMR (CDCl_3_): *δ* = 7.52 (d, *J* = 8.0 Hz, 1H), 7.42-7.31 (m, 2H), 3.92 (s, 3H), 3.90 (s, 3H).^13^C-NMR (CDCl_3_): δ = 166.1, 159.2, 132.8, 129.5, 121.6, 119.5, 100.1, 56.3, 52.2.

******: To a 100 mL three neck round-bottomed flask equipped with a stir bar was added methyl 4-iodo-2-methoxy benzoate (0.69 g, 2.4 mmol), bis(pinacolato)diboron (0.72 g, 2.8 mmol), KOAc (0.72 g, 7.1 mmol) and Pd(dppf)Cl_2_ (50 mg). Then 1,4-dioxine (20 mL) was added under N_2_ atmosphere, and the mixture was heated at 90 ^o^C overnight. After cooling down, the mixture was concentrated. The residue was dissolved in EtOAc, washed with brine, dried over Na_2_SO_4_^,^ and concentrated. The residue was purified by column chromatography (petroleum ether/ethyl acetate = 10:1 to 5:1) to afford the desired product as a light yellow oil. (0.65 g, 95%). ^1^H-NMR (CDCl_3_) *δ* = 7.78 (d, *J* = 7.6 Hz, 1H), 7.42 (dd, *J* = 7.6, 0.7 Hz, 1H), 7.40 (s, 1H), 3.97 (s, 3H), 3.91 (s, 3H), 1.37 (s, 12H). ^13^C-NMR (CDCl_3_) *δ* = 166.8, 158.3, 130.7, 126.5, 122.4, 117.6, 84.2, 56.1, 52.1, 25.0, 24.9.

******: Under an argon atmosphere, methyl 2-methoxy-4-(4,4,5,5-tetramethyl-1,3,2-dioxaborolan-2-yl) benzoate (0.32 g, 1.1 mmol), 4-iodo-2-methoxy benzoate (0.29 g, 1.0 mmol), and Pd(PPh_3_)_4_ (30 mg, 0.03 mmol) were dissolved in 1,4-dioxane/H_2_O = 2:1 (15 mL). A solution of aqueous CsF (2 M, 2.0 mL) was added, and the mixture was stirred at 80 ^o^C overnight. After cooling down, brine (15 mL) was added, and the mixture was extracted with ethyl acetate (2 × 30 mL). The combined organic layers were washed with water, and dried over Na_2_SO_4_. The crude product was purified by column chromatography (petroleum ether/ethyl acetate 10:1 to 4:1) to afford the title compound as a white solid (0.28 g, 85%). ^1^H-NMR (CDCl_3_): *δ* = 7.92 (d, *J* = 7.9 Hz, 2H), 7.22 (d, *J* = 9.1 Hz, 2H), 7.17 (s, 2H), 4.01 (s, 6H), 3.94 (s, 6H). ^13^C-NMR (CDCl_3_): 166.4. 159.6, 145.7, 132.4, 119.5, 119.2, 111.0, 56.2, 52.2.

: LiAlH_4_ (76 mg, 2 mmol) was added to a solution of dimethyl 3,3'-dimethoxy-[1,1'-biphenyl]-4,4'-dicarboxylate (0.17 g，0.5 mmol) in freshly distilled THF (10 mL) stirred at 0 ℃ in three portions during 15 minutes. The mixture was heated at reflux for 3 h. After cooling down to room temperature, 2 mL water was added to the solution, and the mixture was extracted with EtOAc (3 × 20 mL). The combined organic layers were washed with brine, and dried over Na_2_SO_4_. The crude product was used for the next step without further purification.

PCC (0.37 g, 1.65 mmol) was added to the solution of above product in DCM (20 mL). The mixture was stirred at room temperature until the TLC showed the reaction was completed. After filtration through a pad of Celite the solvent was evaporated, and the residue was purified by column chromatography (petroleum ether/ethyl acetate 10:1 to 3:1) to afford the title compound as a white solid (0.12 g, 80%). ^1^H-NMR (CDCl_3_): *δ* = 10.53 (s, 2H), 7.95 (d, *J* = 8.0 Hz, 2H), 7.27 (d, *J* = 3.9 Hz, 2H), 7.19 (s, 2H), 4.05 (s, 6H). ^13^C-NMR (CDCl_3_): 189.3, 162.1, 147.8, 129.2, 124.5, 119.9, 110.6, 55.9.

******: BBr_3_ (1.0 M in CH_2_Cl_2_, 10 mL) was added dropwise to a solution of 3,3’-dimethoxy-[1,1’-biphenyl]-4,4’-dicarbaldehyde (1.0 g, 3.7 mmol) in anhydrous DCM (20 mL) with stirring at 0 ℃ under N_2_ atmosphere. Ice water was added after TLC indicated the reaction completed. The mixture was extracted with EtOAc (3 × 20 mL), and the combined organic layers were washed with 5% aqueous NaHCO_3_ and brine, dried over Na_2_SO_4_, and evaporated. The crude product was purified by column chromatography (petroleum ether/ethyl acetate 10:1 to 4:1) to afford the desired compound as a white solid (0.80 g, 90%). ^1^H-NMR (CDCl_3_): *δ* = 11.13 (s, 2H), 9.98 (s, 2H), 7.69 (d, *J* = 7.9 Hz, 2H), 7.29 (d, *J* = 6.0 Hz, 2H) , 7.26 (s, 2H); ^13^C-NMR (CDCl_3_): 196.1, 161.8, 147.8, 134.2, 120.4, 119.0, 116.5. ESI-MS (*m*/*z*) calcd for C_14_H_11_O_4_ (M+H^+^): 243.1; found: 243.1.­­_­_

******: It was prepared from 5-hydroxyisophthalic acid in three steps according to the literature procedure.^1^

******: This compound was obtained according to the literature procedure.^2^ The data was well consistent with the literature.

: It was synthesized in two steps from 5-bromo-2-methyl-pyridine using a reported method.^3^

: It was synthesized in two steps from 4,4'-dimethoxy-1,1'-biphenyl using a reported method.^4^

1. **^1^H-NMR and ^13^C-NMR**


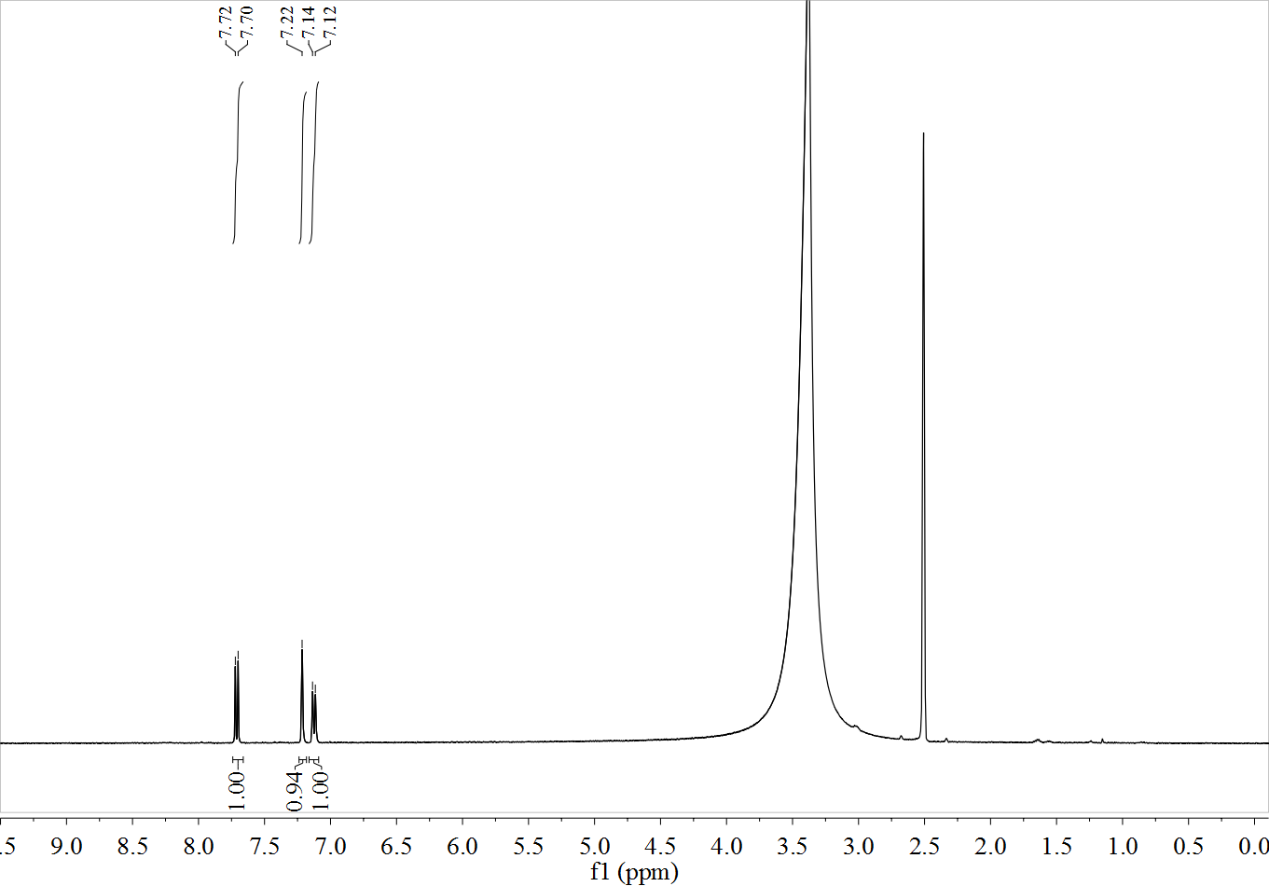


**Figure S1**. ^1^H-NMR of 2-hydroxy-4-iodobenzoic acid. Solvent: DMSO-*d*_6_

**Figure S2**. ^1^H-NMR of methyl 4-iodo-2-methoxybenzoate. Solvent: CDCl_3_


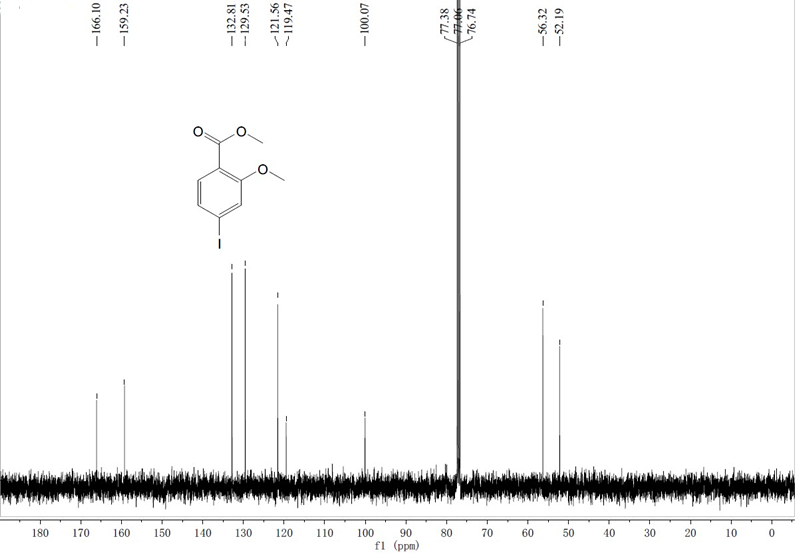


**Figure S3**. ^13^C-NMR of methyl 4-iodo-2-methoxybenzoate. Solvent: CDCl_3_


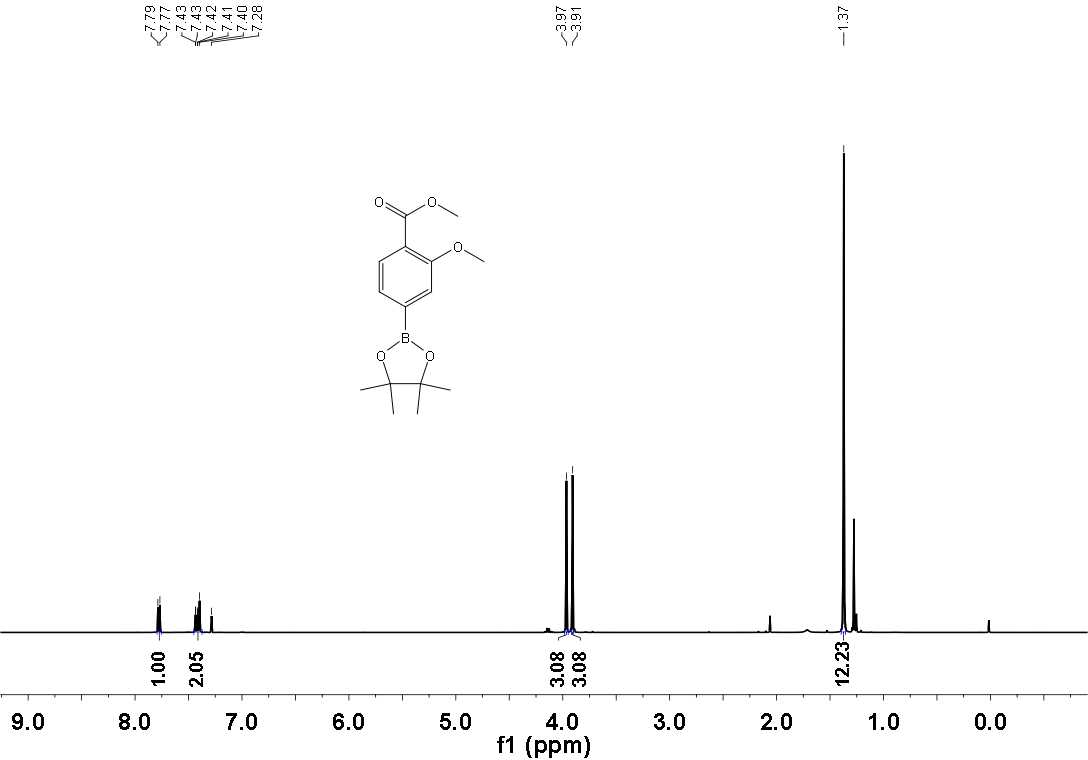


**Figure S4**. ^1^H-NMR of 3-Methoxy-4-(methoxycarbonyl)phenylboronic acid pinacol ester. Solvent: CDCl_3_


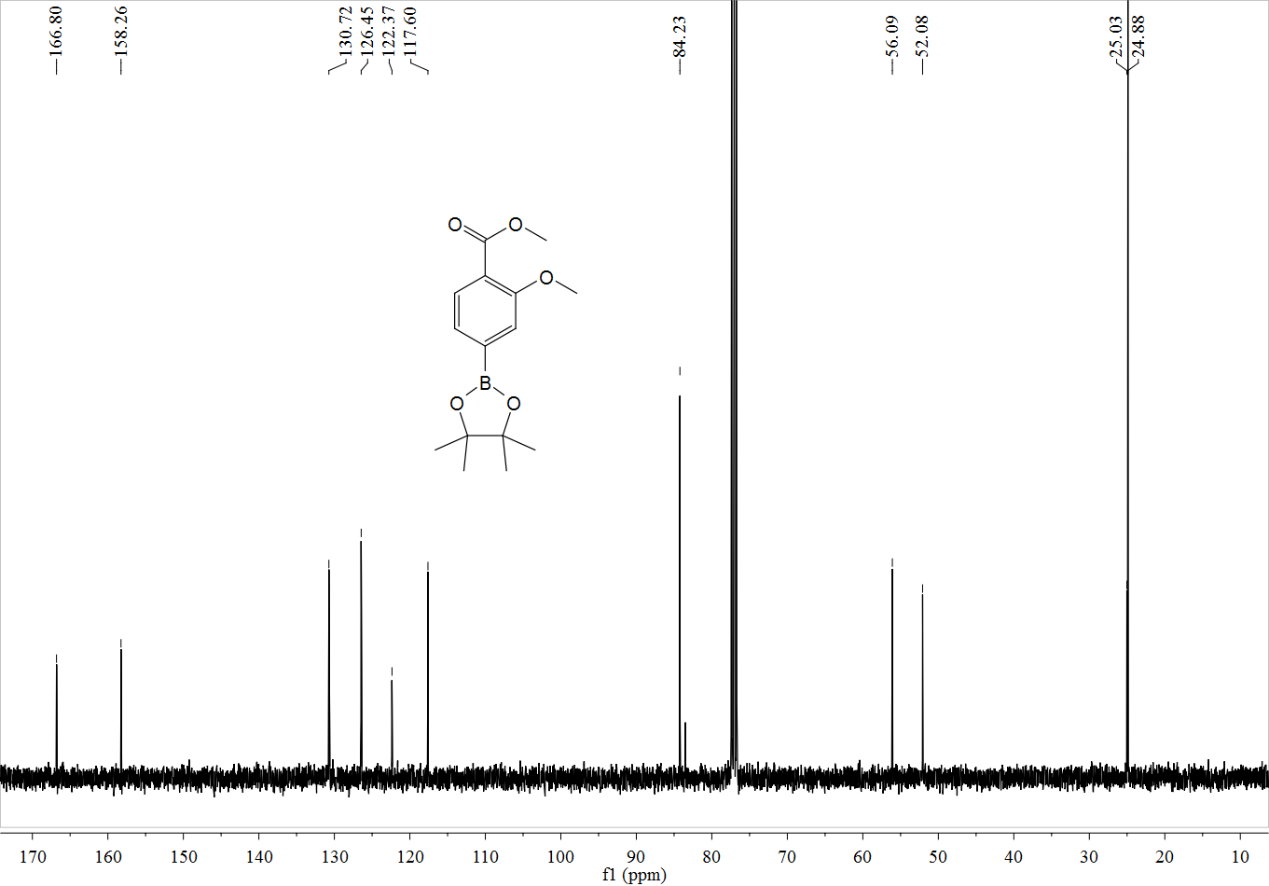


**Figure S5**. ^13^C-NMR of 3-Methoxy-4-(methoxycarbonyl)phenylboronic acid pinacol ester. Solvent: CDCl_3_

 **Figure S6**. ^1^H-NMR of dimethyl 3,3'-dimethoxy-[1,1'-biphenyl]-4,4'-dicarboxylate. Solvent: CDCl_3_


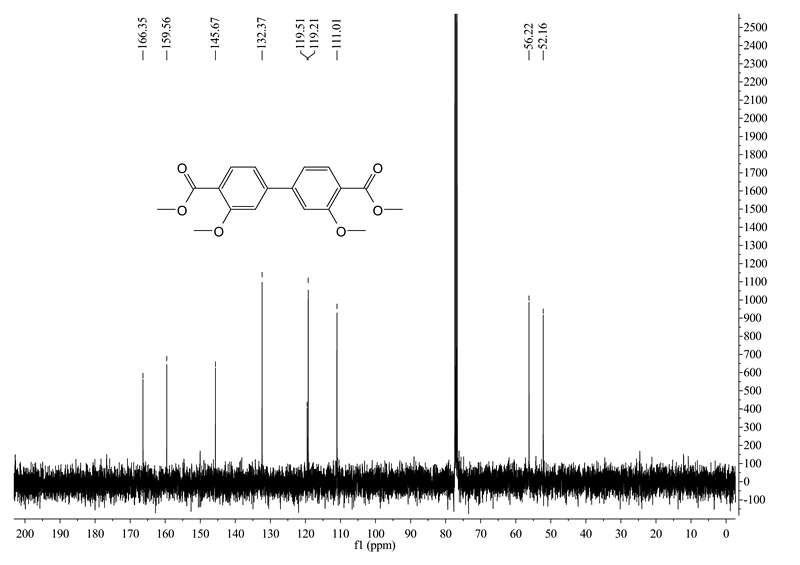


**Figure S7**. ^13^C-NMR of dimethyl 3,3'-dimethoxy-[1,1'-biphenyl]-4,4'-dicarboxylate. Solvent: CDCl_3_

**Figure S8**. ^1^H-NMR of 3,3'-dimethoxy-[1,1'-biphenyl]-4,4'-dicarbaldehyde. Solvent: CDCl_3_

**
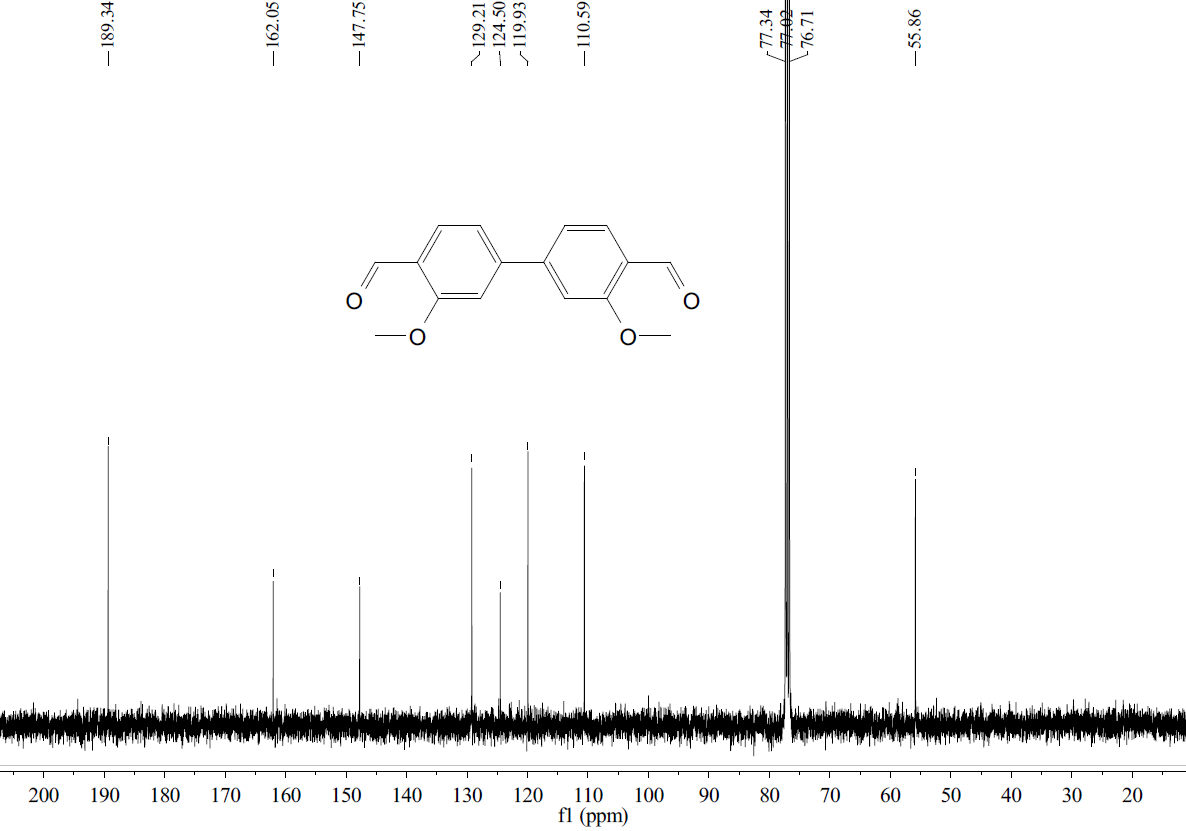
**

**Figure S9**. ^13^C-NMR of 3,3'-dimethoxy-[1,1'-biphenyl]-4,4'-dicarbaldehyde. Solvent: CDCl_3_

**Figure S10**. ^1^H-NMR of 3,3'-dihydroxy-[1,1'-biphenyl]-4,4'-dicarbaldehyde. Solvent: CDCl_3_


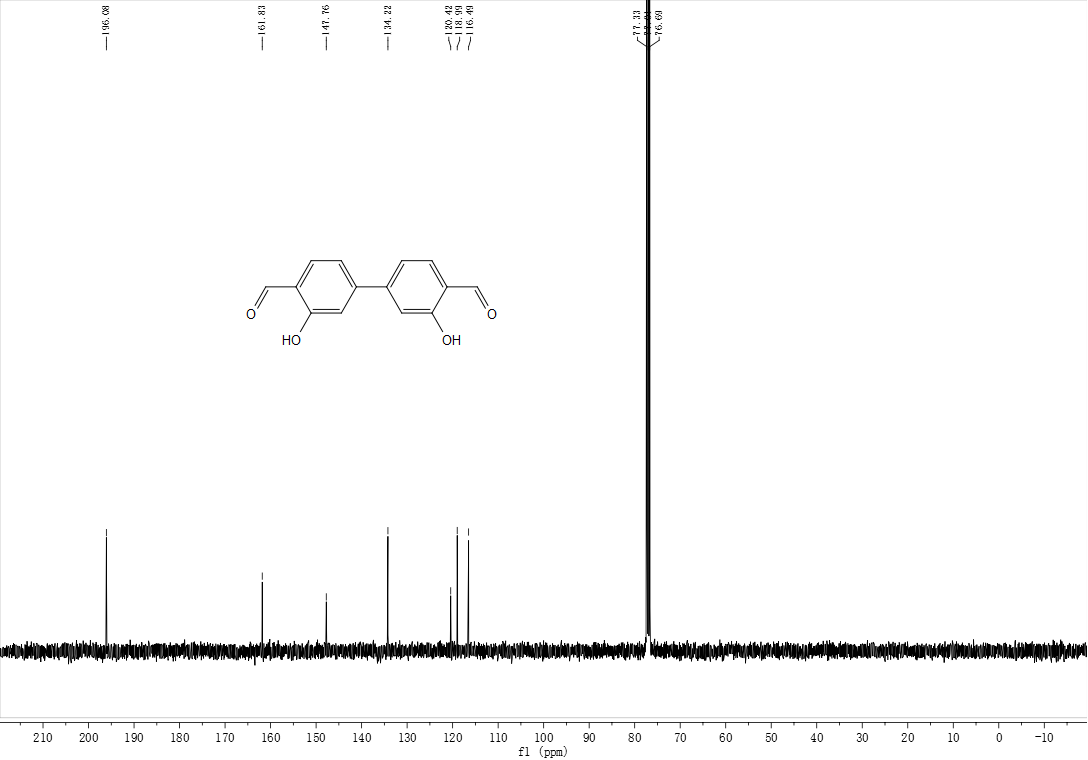


**Figure S11**. ^13^C-NMR of 3,3'-dihydroxy-[1,1'-biphenyl]-4,4'-dicarbaldehyde. Solvent: CDCl_3_


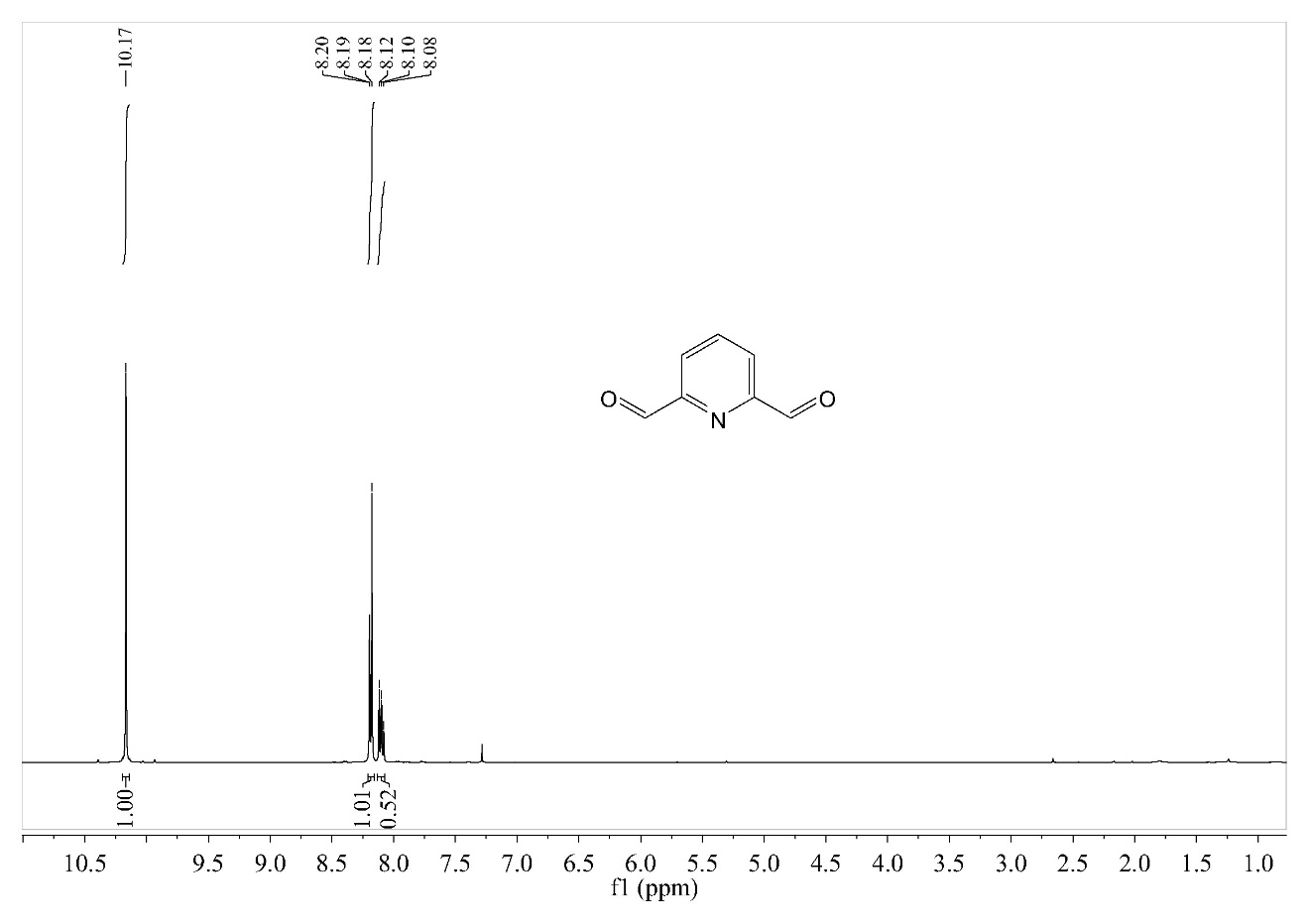


**Figure S12**. ^1^H-NMR of pyridine-2,6-dicarbaldehyde. Solvent: CDCl_3_


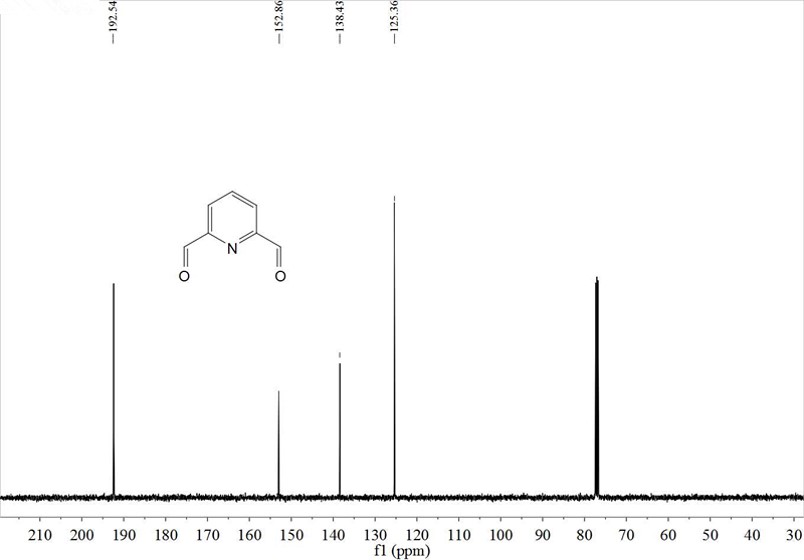


**Figure S13**. ^13^C-NMR of pyridine-2,6-dicarbaldehyde. Solvent: CDCl_3_


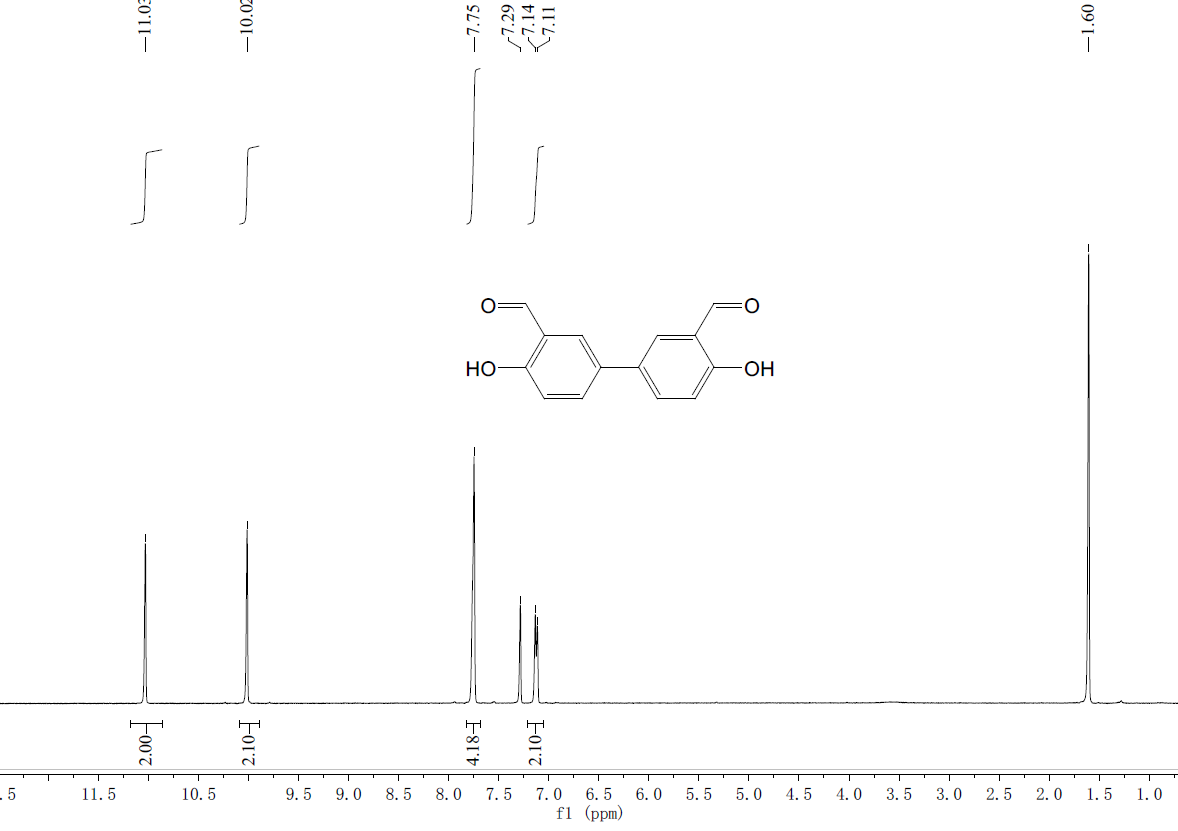


**Figure S14**. ^1^H-NMR of 4,4'-dihydroxy-[1,1'-biphenyl]-3,3'-dicarbaldehyde. Solvent: CDCl_3_


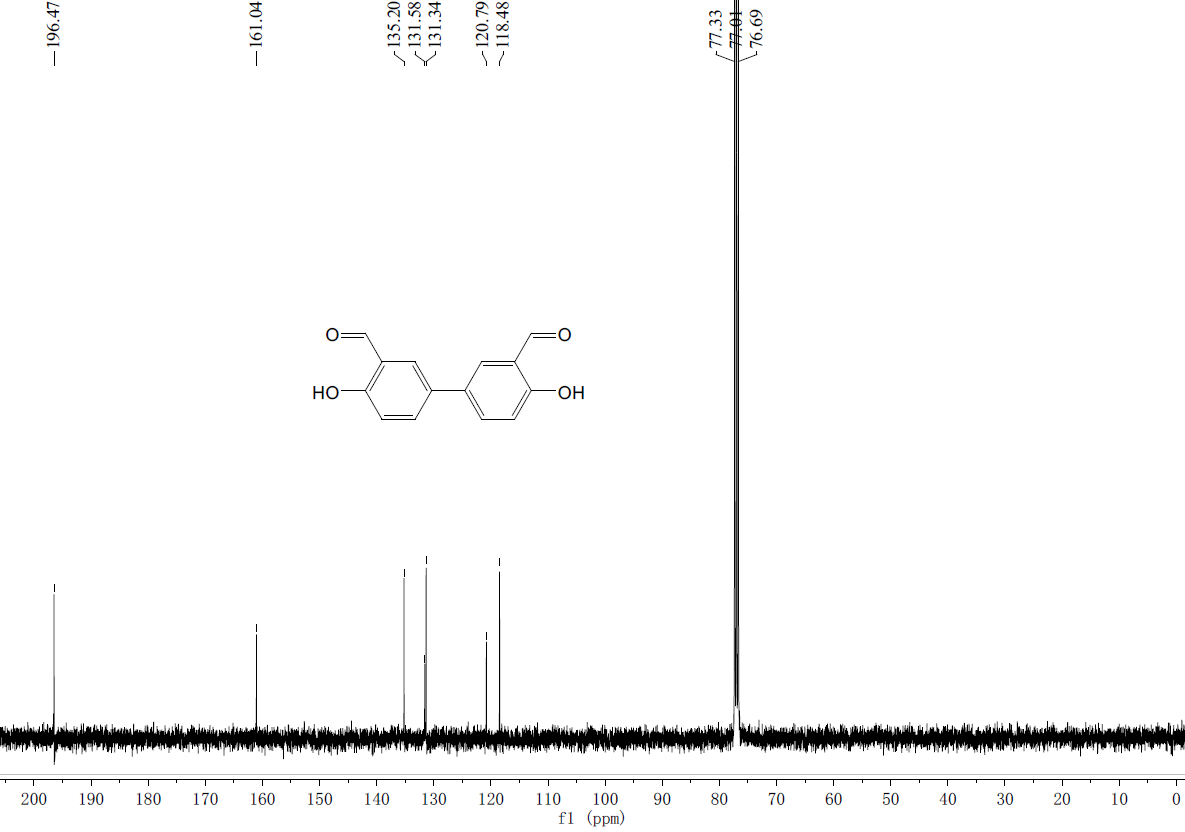


**
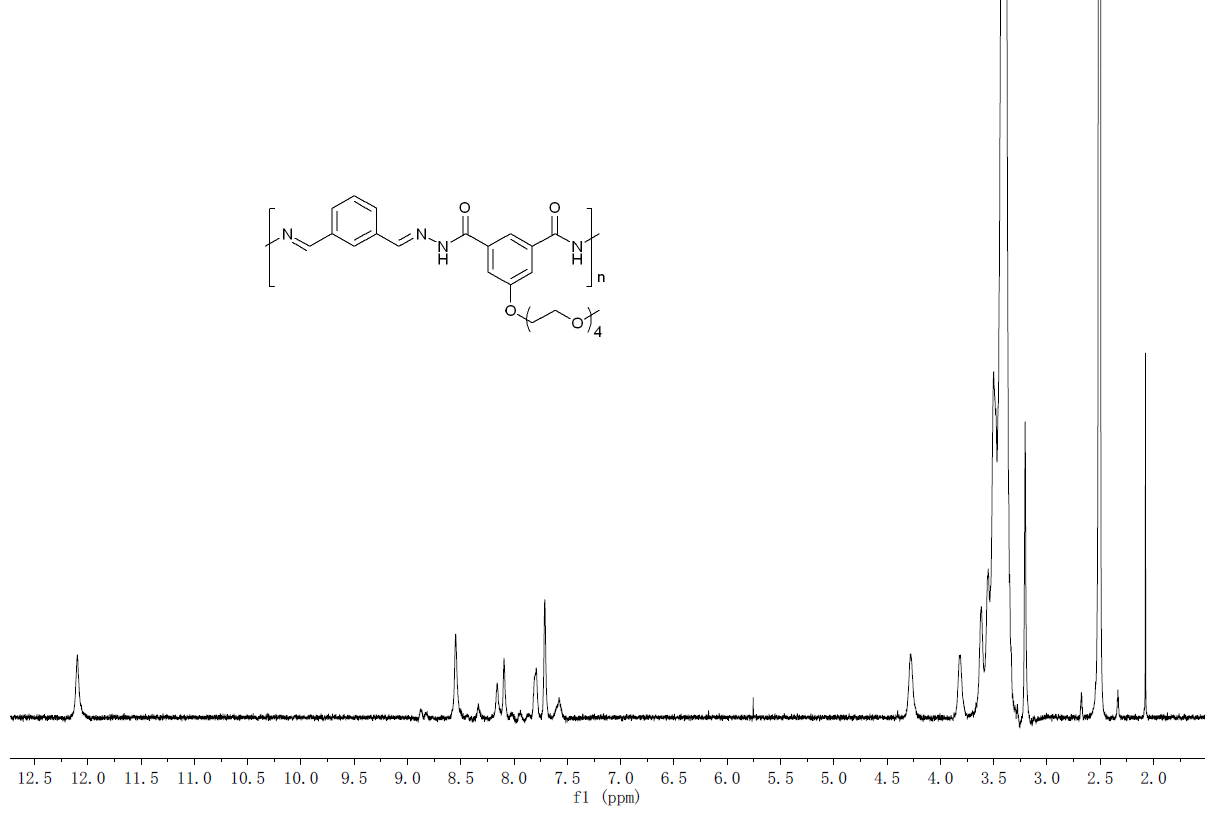
Figure S15**. ^13^C-NMR of 4,4'-dihydroxy-[1,1'-biphenyl]-3,3'-dicarbaldehyde. Solvent: CDCl_3_

**Figure S16.** ^1^H-NMR spectrum of **P1**. Solvent: DMSO-*d*_6_


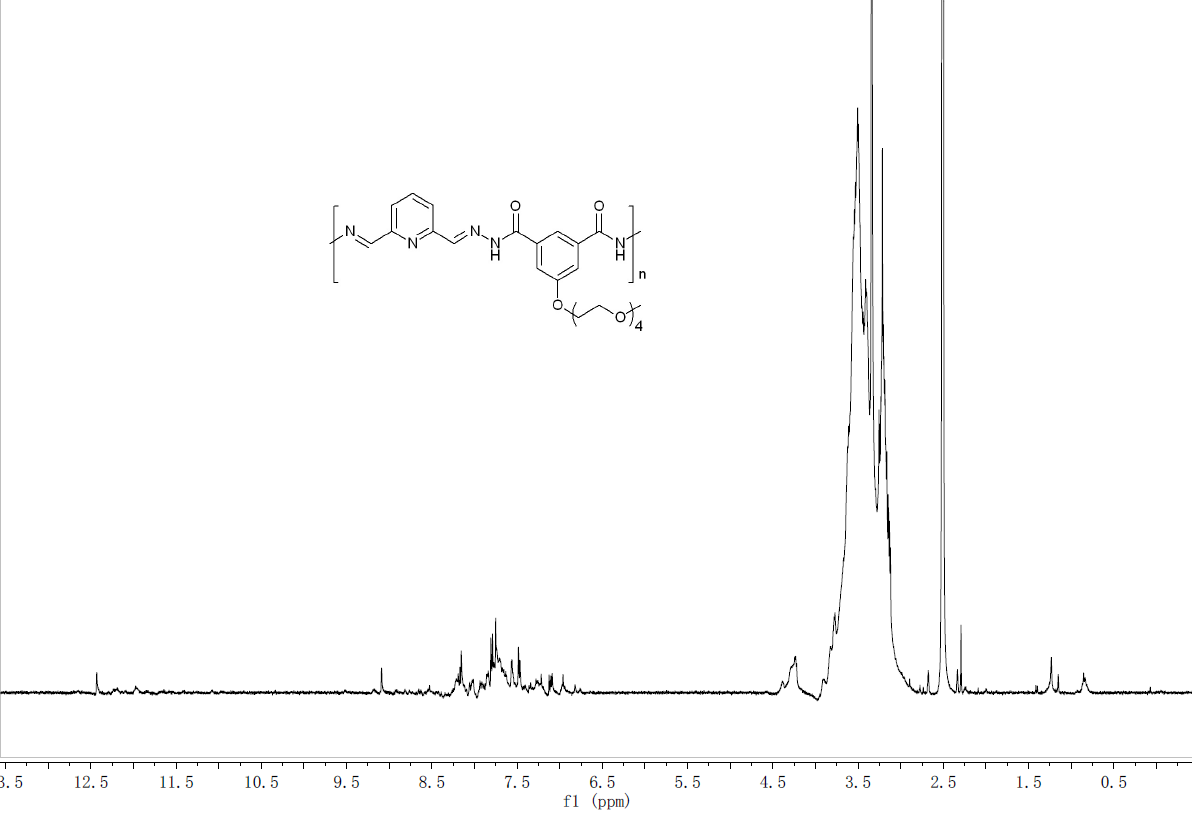
**Figure S17**. ^1^H-NMR spectrum of **P2**. Solvent: DMSO-*d*_6_

_
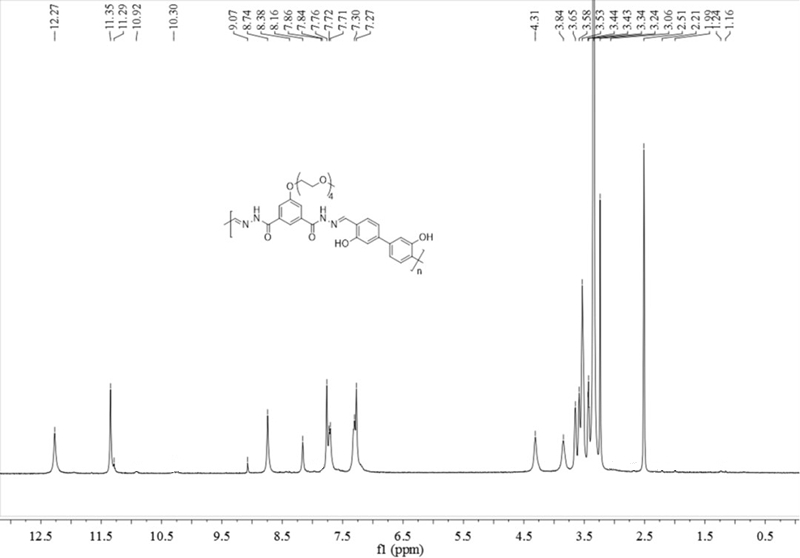
_

**Figure S18**. ^1^H-NMR spectrum of **P3**. Solvent: DMSO-*d*_6_

_
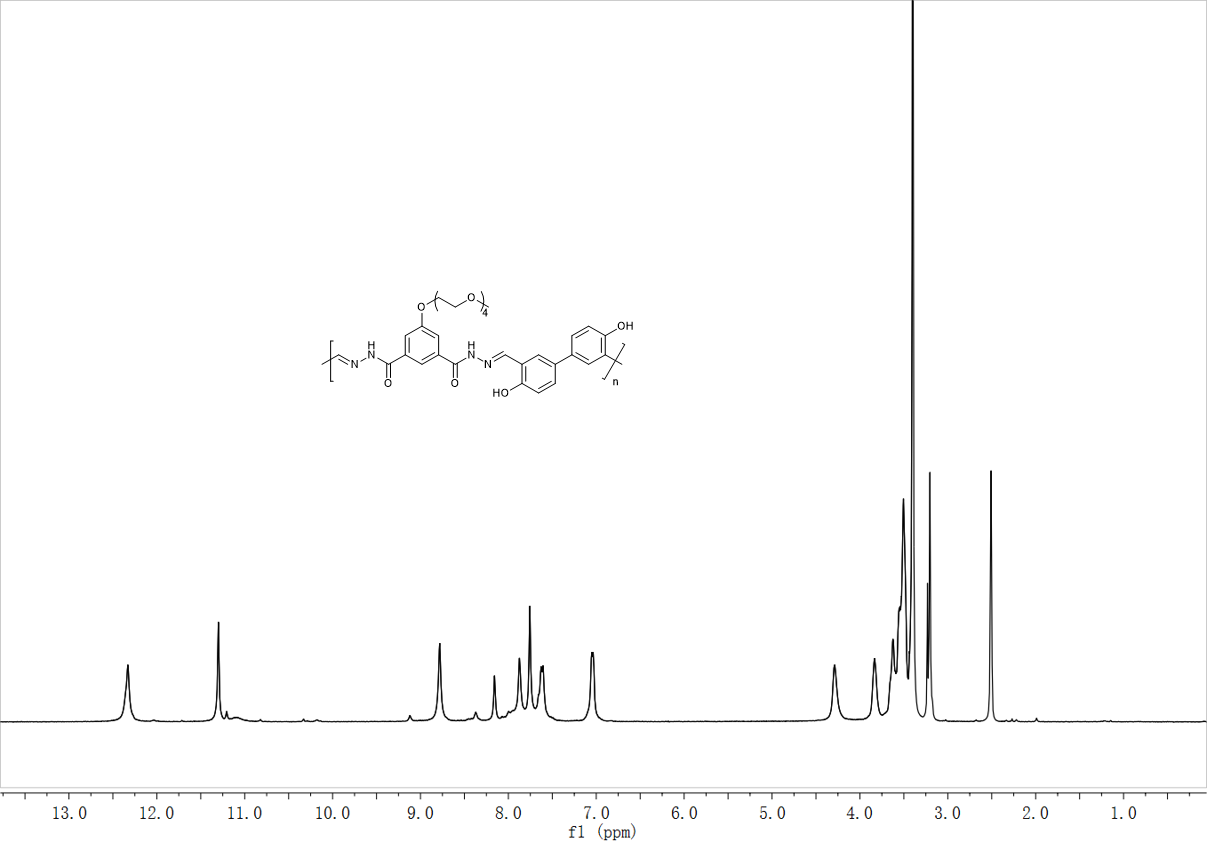
_

**Figure S19**. ^1^H-NMR spectrum of **P4**. Solvent: DMSO-*d*_6_

**Figure S20**. ^1^H-NMR spectrum of **P5**. Solvent: DMSO-*d*_6_

- 1. **GPC Data**


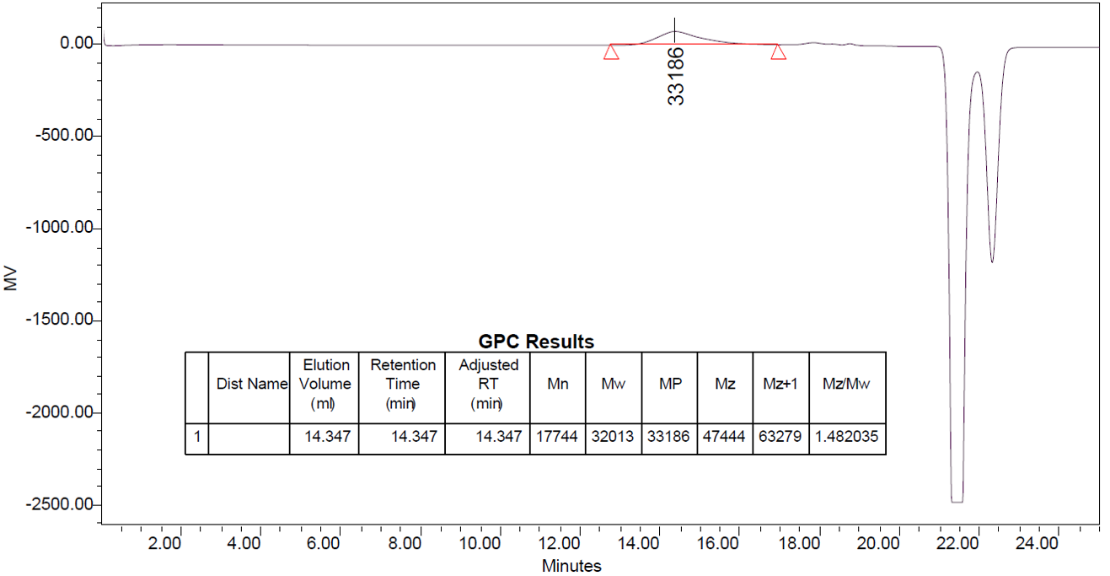


**Figure S21**. GPC data for **P1**.


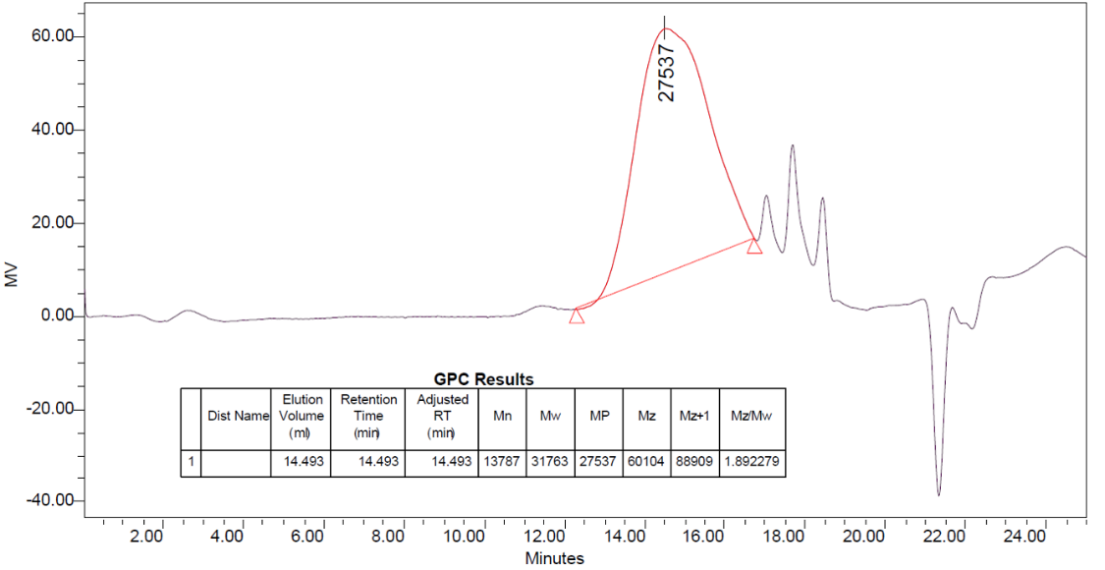


**Figure S22**. GPC data for **P2**.


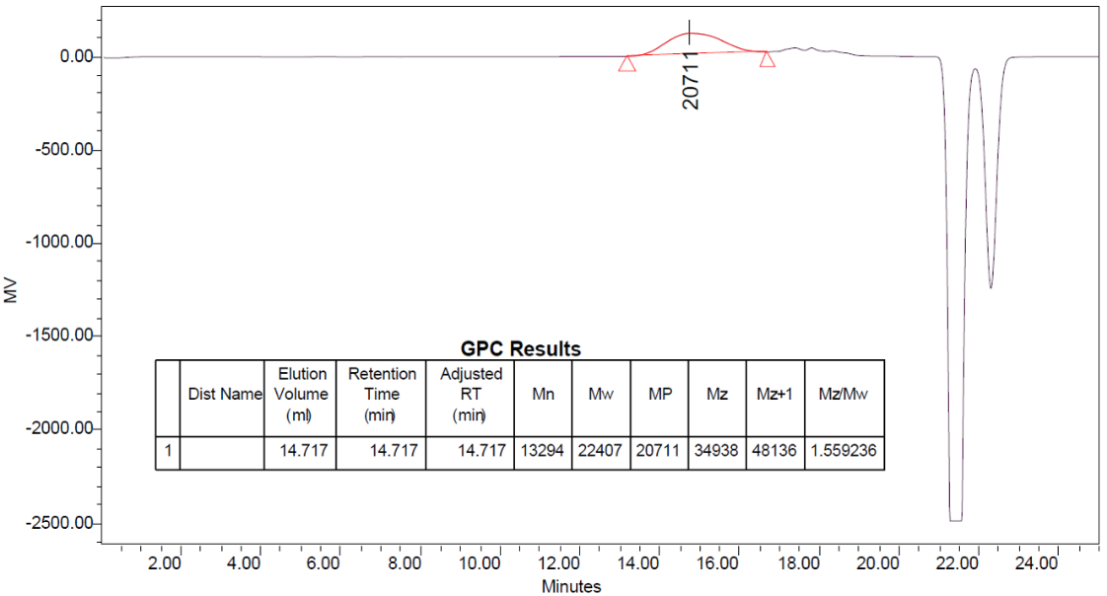


**Figure S23**. GPC data for **P3**.

_
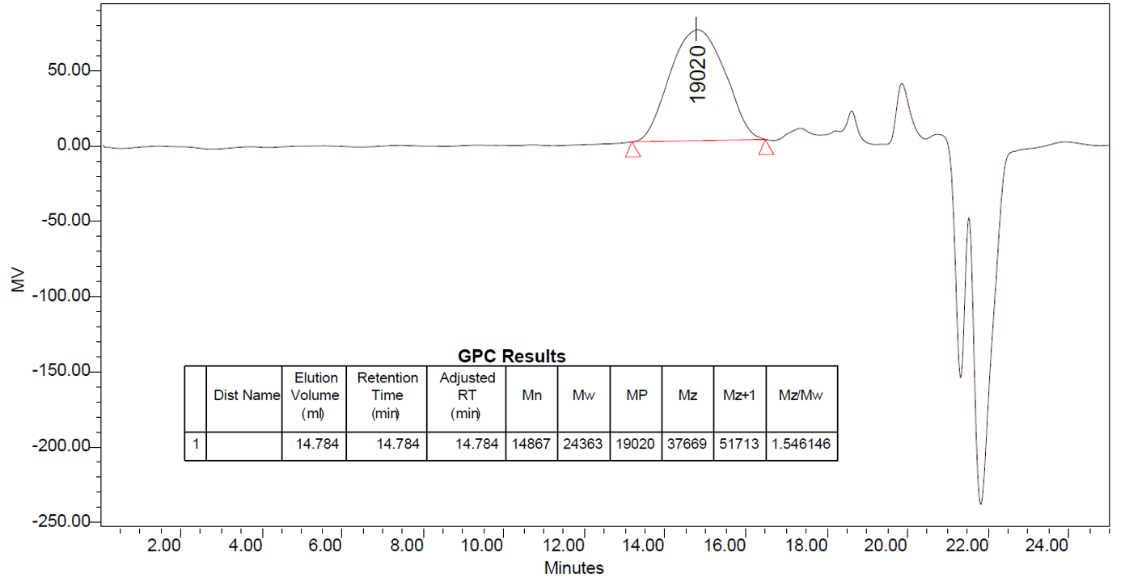
_

**Figure S24**. GPC data for **P4**.

1. **Spectroscopic Data**
   1. **Polymer 1 in DMSO Solution**

**Figure S25**. UV-vis absorption spectra of **P1** (15.2 μg/mL) with addition of various concentrations of Zn(OTf)_2_ (0 to 51.36 μM) in 3.0 mL DMSO solution.

**Figure S26**. UV-vis absorption spectra of **P1** (15.2 μg/mL) with addition of various concentrations of Ni(ClO_4_)_2_ (0 to 87.17 μM) in 3.0 mL DMSO solution.

**Figure S27**. UV-vis absorption spectra of **P1** (15.2 μg/mL) with addition of various concentrations of Cd(ClO_4_)_2_ (0 to 71.71 μM) in 3.0 mL DMSO solution.

**Figure S28**. UV-vis absorption spectra of **P1** (15.2 μg/mL) with addition of various concentrations of Mn(ClO_4_)_2_ (0 to 76.93 μM) in 3.0 mL DMSO solution.

**Figure S29**. UV-vis absorption spectra of **P1** (15.2 μg/mL) with addition of various concentrations of La(OTf)_3_ (0 to 89.54 μM) in 3.0 mL DMSO solution.

**Figure S30**. UV-vis absorption spectra of **P1** (15.2 μg/mL) with addition of various concentrations of Bu_4_NF (0 to 39.95 μM) in 3.0 mL DMSO solution.

**Figure S31**. UV-vis absorption spectra of **P1** (15.2 μg/mL) with addition of various concentrations of PPi (0 to 39.85 μM) in 3.0 mL DMSO solution.

**Figure S32**. UV-vis absorption spectra of **P1** (15.2 μg/mL) with addition of various concentrations of Bu_4_NOTf (0 to 423.63 μM) in 3.0 mL DMSO solution.

- 1. **Polymer 1 in DMSO : H_2_O (1 : 1 v/v) Solution**

**Figure S33**. UV-vis absorption spectra of **P1** (15.2 μg/mL) with addition of various concentrations of Zn(OTf)_2_ (0 to 37.60 μM) in 3.0 mL DMSO : H_2_O (1 : 1 v/v) solution.

**Figure S34**. UV-vis absorption spectra of **P1** (15.2 μg/mL) with addition of various concentrations of Ni(ClO_4_)_2_ (0 to 27.83 μM) in 3.0 mL DMSO : H_2_O (1 : 1 v/v) solution.

**Figure S35**. UV-vis absorption spectra of **P1** (15.2 μg/mL) with addition of various concentrations of Cd(ClO_4_)_2_ (0 to 71.71 μM) in 3.0 mL DMSO : H_2_O (1 : 1 v/v) solution.

**Figure S36**. UV-vis absorption spectra of **P1** (15.2 μg/mL) with addition of various concentrations of Mn(ClO_4_)_2_ (0 to 76.93 μM) in 3.0 mL DMSO : H_2_O (1 : 1 v/v) solution.

**Figure S37**. UV-vis absorption spectra of **P1** (15.2 μg/mL) with addition of various concentrations of La(OTf)_3_ (0 to 89.54 μM) in 3.0 mL DMSO : H_2_O (1 : 1 v/v) solution.

- 1. **Polymer 2 in DMSO Solution**

**Figure S38**. UV-vis absorption spectra of **P2** (17.8 μg/mL) with addition of various concentrations of Zn(OTf)_2_ (0 to 265.95 μM) in 3.0 mL DMSO solution.

**Figure S39**. UV-vis absorption spectra of **P2** (17.8 μg/mL) with addition of various concentrations of Ni(ClO_4_)_2_ (0 to 93.39 μM) in 3.0 mL DMSO solution.

**Figure S40**. UV-vis absorption spectra of **P2** (17.8 μg/mL) with addition of various concentrations of Cd(ClO_4_)_2_ (0 to 61.91 μM) in 3.0 mL DMSO solution.

**Figure S41**. UV-vis absorption spectra of **P2** (17.8 μg/mL) with addition of various concentrations of Mn(ClO_4_)_2_ (0 to 56.73 μM) in 3.0 mL DMSO solution.

**Figure S42**. UV-vis absorption spectra of **P2** (17.8 μg/mL) with addition of various concentrations of La(OTf)_3_ (0 to 89.94 μM) in 3.0 mL DMSO solution.

**Figure S43**. UV-vis absorption spectra of **P2** (17.8 μg/mL) with addition of various concentrations of Bu_4_NF (0 to 269.95 μM) in 3.0 mL DMSO solution.

**Figure S44**. UV-vis absorption spectra of **P2** (17.8 μg/mL) with addition of various concentrations of PPi (0 to 98.31 μM) in 3.0 mL DMSO solution.

- 1. **Polymer 2 in DMSO : H_2_O (1 : 1 v/v) Solution**

**Figure S45**. UV-vis absorption spectra of **P2** (17.8 μg/mL) with addition of various concentrations of Zn(OTf)_2_ (0 to 30.17 μM) in 3.0 mL DMSO : H_2_O (1 : 1 v/v) solution.

**Figure S46**. UV-vis absorption spectra of **P2** (17.8 μg/mL) with addition of various concentrations of Ni(ClO_4_)_2_ (0 to 80.27 μM) in 3.0 mL DMSO : H_2_O (1 : 1 v/v) solution.

**Figure S47**. UV-vis absorption spectra of **P2** (17.8 μg/mL) with addition of various concentrations of Cd(ClO_4_)_2_ (0 to 81.27 μM) in 3.0 mL DMSO : H_2_O (1 : 1 v/v) solution.

**Figure S48**. UV-vis absorption spectra of **P2** (17.8 μg/mL) with addition of various concentrations of Mn(ClO_4_)_2_ (0 to 90.34 μM) in 3.0 mL DMSO : H_2_O (1 : 1 v/v) solution.

**Figure S49**. UV-vis absorption spectra of **P2** (17.8 μg/mL) with addition of various concentrations of La(OTf)_3_ (0 to 90.14 μM) in 3.0 mL DMSO : H_2_O (1 : 1 v/v) solution.

**Figure S50**. UV-vis absorption spectra of **P2** (17.8 μg/mL) with addition of various concentrations of Bu_4_NF (0 to 95.38 μM) in 3.0 mL DMSO : H_2_O (1 : 1 v/v) solution.

**Figure S51**. UV-vis absorption spectra of **P2** (17.8 μg/mL) with addition of various concentrations of PPi (0 to 85.13 μM) in 3.0 mL DMSO : H_2_O (1 : 1 v/v) solution.

- 1. **Polymer 3 in DMSO Solution**

**Figure S52**. UV-vis absorption spectra of **P3** (7.97 μg/mL) with addition of various concentrations of Zn(OTf)_2_ (0 to 98.31 μM) in 3.0 mL DMSO solution.

**Figure S53**. UV-vis absorption spectra of **P3** (7.97 μg/mL) with addition of various concentrations of Ni(ClO_4_)_2_ (0 to 168.42 μM) in 3.0 mL DMSO solution.

**Figure S54**. UV-vis absorption spectra of **P3** (7.97 μg/mL) with addition of various concentrations of Cd(ClO_4_)_2_ (0 to 81.80 μM) in 3.0 mL DMSO solution.

**Figure S55**. UV-vis absorption spectra of **P3** (7.97 μg/mL) with addition of various concentrations of Mn(ClO_4_)_2_ (0 to 288.93 μM) in 3.0 mL DMSO solution.

**Figure S56**. UV-vis absorption spectra of **P3** (7.97 μg/mL) with addition of various concentrations of La(OTf)_3_ (0 to 220.37 μM) in 3.0 mL DMSO solution.

**Figure S57**. UV-vis absorption spectra of **P3** (7.97 μg/mL) with addition of various concentrations of Bu_4_NOAc (0 to 202.82 μM) in 3.0 mL DMSO solution.

**Figure S58**. UV-vis absorption spectra of **P3** (7.97 μg/mL) with addition of various concentrations of Bu_4_NF (0 to 223.63 μM) in 3.0 mL DMSO solution.

**Figure S59**. UV-vis absorption spectra of **P3** (7.97 μg/mL) with addition of various concentrations of PPi (0 to 163.24 μM) in 3.0 mL DMSO solution.

**Figure S60**. UV-vis absorption spectra of **P3** (7.97 μg/mL) with addition of various concentrations of Bu_4_NCl (0 to 754.11 μM) in 3.0 mL DMSO solution.

**Figure S61**. UV-vis absorption spectra of **P3** (7.97 μg/mL) with addition of various concentrations of Bu_4_NBr (0 to 93.11 μM) in 3.0 mL DMSO solution.

**Figure S62**. UV-vis absorption spectra of **P3** (7.97 μg/mL) with addition of various concentrations of Bu_4_NOTf (0 to 236.11 μM) in 3.0 mL DMSO solution.

- 1. **Polymer 3 in DMSO : H_2_O (4 : 1 v/v) Solution**

**Figure S63**. UV-vis absorption spectra of **P3** (7.97 μg/mL) with addition of various concentrations of Zn(OTf)_2_ (0 to 540.59 μM) in 3.0 mL DMSO : H_2_O (4 : 1 v/v) solution.

**Figure S64**. UV-vis absorption spectra of **P3** (7.97 μg/mL) with addition of various concentrations of Ni(ClO_4_)_2_ (0 to 419.25 μM) in 3.0 mL DMSO : H_2_O (4 : 1 v/v) solution.

**Figure S65**. UV-vis absorption spectra of **P3** (7.97 μg/mL) with addition of various concentrations of Cd(ClO_4_)_2_ (0 to 168.15 μM) in 3.0 mL DMSO : H_2_O (4 : 1 v/v) solution.

**Figure S66**. UV-vis absorption spectra of **P3** (7.97 μg/mL) with addition of various concentrations of Mn(ClO_4_)_2_ (0 to 661.01 μM) in 3.0 mL DMSO : H_2_O (4 : 1 v/v) solution.

**Figure S67**. UV-vis absorption spectra of **P3** (7.97 μg/mL) with addition of various concentrations of La(OTf)_3_ (0 to 266.55 μM) in 3.0 mL DMSO : H_2_O (4 : 1 v/v) solution.

**Figure S68**. UV-vis absorption spectra of **P3** (7.97 μg/mL) with addition of various concentrations of Bu_4_NOAc (0 to 478.19 μM) in 3.0 mL DMSO : H_2_O (4 : 1 v/v) solution.

**Figure S69**. UV-vis absorption spectra of **P3** (7.97 μg/mL) with addition of various concentrations of Bu_4_NF (0 to 231.24 μM) in 3.0 mL DMSO : H_2_O (4 : 1 v/v) solution.

**Figure S70**. UV-vis absorption spectra of **P3** (7.97 μg/mL) with addition of various concentrations of PPi (0 to 692.55 μM) in 3.0 mL DMSO : H_2_O (4 : 1 v/v) solution.

- 1. **Polymer 3 in DMSO : H_2_O (1 : 1 v/v) Solution**

**Figure S71**. UV-vis absorption spectra of **P3** (7.97 μg/mL) with addition of various concentrations of Zn(OTf)_2_ (0 to 93.87 μM) in 3.0 mL DMSO : H_2_O (1 : 1 v/v) solution.

**Figure S72**. UV-vis absorption spectra of **P3** (7.97 μg/mL) with addition of various concentrations of Ni(OTf)_2_ (0 to 298.44 μM) in 3.0 mL DMSO : H_2_O (1 : 1 v/v) solution.

**Figure S73**. UV-vis absorption spectra of **P3** (7.97 μg/mL) with addition of various concentrations of Cd(ClO_4_)_2_ (0 to 63.21 μM) in 3.0 mL DMSO : H_2_O (1 : 1 v/v) solution.

**Figure S74**. UV-vis absorption spectra of **P3** (7.97 μg/mL) with addition of various concentrations of Mn(ClO_4_)_2_ (0 to 63.21 μM) in 3.0 mL DMSO : H_2_O (1 : 1 v/v) solution.

**Figure S75**. UV-vis absorption spectra of **P3** (7.97 μg/mL) with addition of various concentrations of La(OTf)_3_ (0 to 103.34 μM) in 3.0 mL DMSO : H_2_O (1 : 1 v/v) solution.

- 1. **Polymer 4 in DMSO Solution**

**Figure S76**. UV-vis absorption spectra of **P4** (9.37 μg/mL) with addition of various concentrations of Zn(OTf)_2_ (0 to 54.22 μM) in 3.0 mL DMSO solution.

**Figure S77**. UV-vis absorption spectra of **P4** (9.37 μg/mL) with addition of various concentrations of Ni(ClO_4_)_2_ (0 to 80.21 μM) in 3.0 mL DMSO solution.

**Figure S78**. UV-vis absorption spectra of **P4** (9.37 μg/mL) with addition of various concentrations of Cd(ClO_4_)_2_ (0 to 64.25 μM) in 3.0 mL DMSO solution.

**Figure S79**. UV-vis absorption spectra of **P4** (9.37 μg/mL) with addition of various concentrations of Mn(ClO_4_)_2_ (0 to 50.11 μM) in 3.0 mL DMSO solution.

**Figure S80**. UV-vis absorption spectra of **P4** (9.37 μg/mL) with addition of various concentrations of La(OTf)_3_ (0 to 60.43 μM) in 3.0 mL DMSO solution.

- 1. **Polymer 5 in DMSO Solution**

**Figure S81**. UV-Vis absorption spectra of **P5** (10.6 μg/mL) with addition of various concentrations of Zn(OTf)_2_ (0 to 7.33 μM) in 2.4 mL DMSO solution.

**Figure S82**. UV-Vis absorption spectra of **P5** (10.6 μg/mL) with addition of various concentrations of Ni(ClO_4_)_2_ (0 to 12.11 μM) in 2.4 mL DMSO solution.

**Figure S83**. UV-Vis absorption spectra of **P5** (10.6 μg/mL) with addition of various concentrations of Cd(ClO_4_)_2_ (0 to 17.18 μM) in 2.4 mL DMSO solution.

**Figure S84**. UV-Vis absorption spectra of **P5** (10.6 μg/mL) with addition of various concentrations of Mn(ClO_4_)_2_ (0 to 49.86 μM) in 2.4 mL DMSO solution.

**Figure S85**. UV-Vis absorption spectra of **P5** (10.6 μg/mL) with addition of various concentrations of La(OTf)_3_ (0 to 15.41 μM) in 2.4 mL DMSO solution.

- 1. **Polymer 5 in DMSO : H_2_O (4 : 1 v/v) Solution**

**Figure S86**. UV-Vis absorption spectra of **P5** (10.6 μg/mL) with addition of various concentrations of Zn(OTf)_2_ (0 to 7.33 μM) in 2.4 mL DMSO : H_2_O (4 : 1 v/v) solution.

**Figure S87**. UV-Vis absorption spectra of **P5** (10.6 μg/mL) with addition of various concentrations of Ni(ClO_4_)_2_ (0 to 10.28 μM) in 2.4 mL DMSO : H_2_O (4 : 1 v/v) solution.

**Figure S88**. UV-Vis absorption spectra of **P5** (10.6 μg/mL) with addition of various concentrations of Cd(ClO_4_)_2_ (0 to 9.12 μM) in 2.4 mL DMSO : H_2_O (4 : 1 v/v) solution.

**Figure S89**. UV-Vis absorption spectra of **P5** (10.6 μg/mL) with addition of various concentrations of Mn(ClO_4_)_2_ (0 to 6.48 μM) in 2.4 mL DMSO : H_2_O (4 : 1 v/v) solution.

**Figure S90**. UV-Vis absorption spectra of **P5** (10.6 μg/mL) with addition of various concentrations of La(OTf)_3_ (0 to 10.50 μM) in 2.4 mL DMSO : H_2_O (4 : 1 v/v) solution.

- 1. **Polymer 5 in DMSO : H_2_O (1 : 1 v/v) Solution**

**Figure S91**. UV-Vis absorption spectra of **P5** (10.6 μg/mL) with addition of various concentrations of Zn(OTf)_2_ (0 to 7.33 μM) in 2.4 mL DMSO : H_2_O (1 : 1 v/v) solution.

**Figure S92**. UV-Vis absorption spectra of **P5** (10.6 μg/mL) with addition of various concentrations of Ni(ClO_4_)_2_ (0 to 9.62 μM) in 2.4 mL DMSO : H_2_O (1 : 1 v/v) solution.

**Figure S93**. UV-Vis absorption spectra of **P5** (10.6 μg/mL) with addition of various concentrations of Cd(ClO_4_)_2_ (0 to 7.41 μM) in 2.4 mL DMSO : H_2_O (1 : 1 v/v) solution.

**Figure S94**. UV-Vis absorption spectra of **P5** (10.6 μg/mL) with addition of various concentrations of Mn(ClO_4_)_2_ (0 to 6.48 μM) in 2.4 mL DMSO : H_2_O (1 : 1 v/v) solution.

**Figure S95**. UV-Vis absorption spectra of **P5** (10.6 μg/mL) with addition of various concentrations of La(OTf)_3_ (0 to 10.52 μM) in 3.0 mL DMSO : H_2_O (1 : 1 v/v) solution.

1. **The Cooperative Effect**

**Figure** **S96**. UV-vis absorption spectra of **P3** (7.97 μg/mL) in the presence of Zn^2+^ (9.8 μg/mL) upon addition of various concentrations of OAc^-^ (0 to 32 μM) in 3.0 mL DMSO solution.

**Figure** **S97**. UV-vis absorption spectra of **P3** (7.97 μg/mL) in the presence of Ni^2+^ (9.8 μg/mL) upon addition of various concentrations of OAc^-^ (0 to 32 μM) in 3.0 mL DMSO solution.

**Figure** **S98**. UV-vis absorption spectra of **P3** (7.97 μg/mL) in the presence of Mn^2+^ (9.8 μg/mL) upon addition of various concentrations of OAc^-^ (0 to 32 μM) in 3.0 mL DMSO solution.

**Figure** **S99**. UV-vis absorption spectra of **P3** (7.97 μg/mL) in the presence of Cd^2+^ (9.8 μg/mL) upon addition of various concentrations of OAc^-^ (0 to 32 μM) in 3.0 mL DMSO solution.

1. **Sensing Application**

********

**Figure S100**. UV-vis absorption spectra of **P2** (17.8 μg/mL) in the presence of Cu^2+^ (4.9 μg/mL) upon addition of various concentrations of 6-MP (0 to 265.95 μM) in 3.0 mL DMSO solution.

**Figure S101**. UV-vis absorption spectra of **P3** (7.97 μg/mL) in the presence of Cu^2+^ (4.9 μg/mL) upon addition of various concentrations of 6-MP (0 to 265.95 μM) in 3.0 mL DMSO solution.

**Figure S102**. UV-vis absorption spectra of **P3** (7.97 μg/mL) in the presence of Cu^2+^ (4.9 μg/mL) upon addition of various concentrations of EDTA (0 to 16.25 μM) in 3.0 mL DMSO solution. Inset: absorbance changes with the addition of EDTA.

**Figure S103**. UV-vis absorption spectra of **P2** (17.8 μg/mL) in the presence of Cu^2+^ (4.9 μg/mL) upon addition of various concentrations of EDTA (0 to 20.81 μM) in 3.0 mL DMSO solution. Inset: absorbance changes with the addition of EDTA.

1. **Reference**

1. D. Zha and L. You, *ACS Appl. Mater. Inter.*, 2016, **8**, 2399-2405.

2. A. Abebayehu, R. Dutta and C. H. Lee, *Chemistry*, 2016, **22**, 13850-13856.

3. W. Meng, T. K. Ronson, J. K. Clegg and J. R. Nitschke, *Angew. Chem. Int. Ed.*, 2013, **52**, 1017-1021.

4. E. Tzur, A. Ben-Asuly, C. E. Diesendruck, I. Goldberg and N. G. Lemcoff, *Angew. Chem. Int. Ed.*, 2008, **47**, 6422-6425.
